# Atlas of quantitative single-base-resolution *N*^6^-methyl-adenine methylomes

**DOI:** 10.1038/s41467-019-13561-z

**Published:** 2019-12-10

**Authors:** Casslynn W. Q. Koh, Yeek Teck Goh, W. S. Sho Goh

**Affiliations:** Genome Institute of Singapore, 60 Biopolis Street, Singapore, 138672 Singapore

**Keywords:** Methylation analysis, Epigenomics

## Abstract

Various methyltransferases and demethylases catalyse methylation and demethylation of *N*^6^-methyladenosine (m6A) and *N*^6^,2′-O-dimethyladenosine (m6Am) but precise methylomes uniquely mediated by each methyltransferase/demethylase are still lacking. Here, we develop m6A-Crosslinking-Exonuclease-sequencing (m6ACE-seq) to map transcriptome-wide m6A and m6Am at quantitative single-base-resolution. This allows for the generation of a comprehensive atlas of distinct methylomes uniquely mediated by every individual known methyltransferase or demethylase. Our atlas reveals METTL16 to indirectly impact manifold methylation targets beyond its consensus target motif and highlights the importance of precision in mapping PCIF1-dependent m6Am. Rather than reverse RNA methylation, we find that both ALKBH5 and FTO instead maintain their regulated sites in an unmethylated steady-state. In FTO’s absence, anomalous m6Am disrupts snRNA interaction with nuclear export machinery, potentially causing aberrant pre-mRNA splicing events.

## Abstract (web summary)

*N*^6^-methyladenosine (m6A) and *N*^6^,2′-O-dimethyladenosine (m6Am) are eukaryotic mRNA modifications. Here the authors develop m6A-Crosslinking-Exonuclease-sequencing to map quantitative methylome changes at single-base-resolution after individually knocking out each known methyltransferase or demethylase.

## Introduction

Out of more than 170 known RNA modifications, *N*^6^-methyladenosine (m6A) is the most abundant mRNA modification. This modification gained prominence after next-generation sequencing mapped m6A transcriptome-wide within mRNAs^1,2^. m6A regulates multiple post-transcriptional processes including but not limited to mRNA decay, mRNA translation, pre-mRNA splicing and pri-miRNA processing^3–7^. Another prominent RNA modification is *N*^6^,2′-O-dimethyladenosine (m6Am), which is formed by the base methylation of 2′-O-methyladenosine (Am) located at the first nucleotide of mRNAs, adjacent to the mRNA cap^8^. m6Am was reported to stabilise mRNA by conferring resistance to DCP2-mediated mRNA-decapping and might also regulate other forms of RNA metabolism^9^.

m6A methyltransferases have been identified in the form of catalytic METTL3 in complex with METTL14, WTAP, KIAA1429 and RBM15/RBM15B^10–16^. The METTL3 methyltransferase complex deposits m6A in the ‘DRm6ACH’ (D = A/G/U, H = A/C/U) motif^17^. However, only a minor fraction of DRACH motifs are methylated, with m6A preferentially localised within the coding DNA sequence (CDS) and 3′ untranslated region (3′UTR) proximal to the stop codon, and to a lesser extent within the 5′UTR. *Mettl3* loss results in a drastic decrease in cellular m6A level, which affects normal cellular differentiation^18,19^. METTL16 is another m6A methyltransferase that directly methylates the ‘UACm6AGAGAA’ motif, though METTL16 depletion also reduces methylation in regions lacking ‘UACAGAGAA’ motifs^20^. Lastly, the discovery of both ALKBH5 and FTO as putative m6A demethylases has suggested that m6A is a dynamic and reversible RNA modification^21–23^. However, no study has yet validated m6A changes at specific sites in response to ALKBH5 or FTO depletion. Furthermore, there are multiple recent reports with conflicting views on the biological function of ALKBH5 and FTO^9,23–29^.

When studying m6A, m6A-RNA-immunoprecpitation-sequencing (m6A-RIP-seq) is the most common m6A-sequencing method utilised but it suffers from poor resolution (~150nt). Single-base-resolution m6A-sequencing techniques have been developed and these involve crosslinking and immunoprecipitating (CLIP) m6A with specific antibodies to induce truncations or mutations at m6A sites during reverse transcription^17,30^. The accuracy of such precise m6A-sequencing techniques has revealed new insights into m6A and m6Am regulation of cellular processes, thereby highlighting the benefits and importance of single-base-resolution m6A sequencing^5,9,16,26^. However, these single-base-resolution techniques are generally time-consuming and involve inconvenient procedures such as radioactive gel electrophoresis. Furthermore, they do not include the use of methylated spike-in controls to correct for antibody immunoprecipitation efficiency, or a RNA input library prepared in parallel for normalisation. Consequently, these techniques are not suitable for quantifying differential methylation between different sample types. This might explain why to date, no effort has been made to precisely map the methylomes specifically mediated by every individual known methyltransferase and demethylase.

In order to overcome the technical limitations of past methods, we developed a novel technique, m6A-Crosslinking-Exonuclease-sequencing (m6ACE-seq) for quantitative single-base-resolution sequencing of m6A and m6Am. We used m6ACE-seq to quantitatively map precise locations of transcriptome-wide m6A/m6Am in cells individually depleted of each and every known catalytic methyltransferase or demethylase, generating a comprehensive atlas of m6A/m6Am methylomes that are regulated by each specific methyltransferase or demethylase. Comparisons of distinct methylomes revealed multiple insights into the regulation and function of m6A and m6Am. Most notably, we redefined FTO as a suppressor of disruptive RNA methylation that can disrupt downstream RNA processing activities, thereby highlighting the utility of our technique in epitranscriptomic studies.

## Results

### m6ACE enriches for RNA fragments starting with m6A

**Fig. 1:**
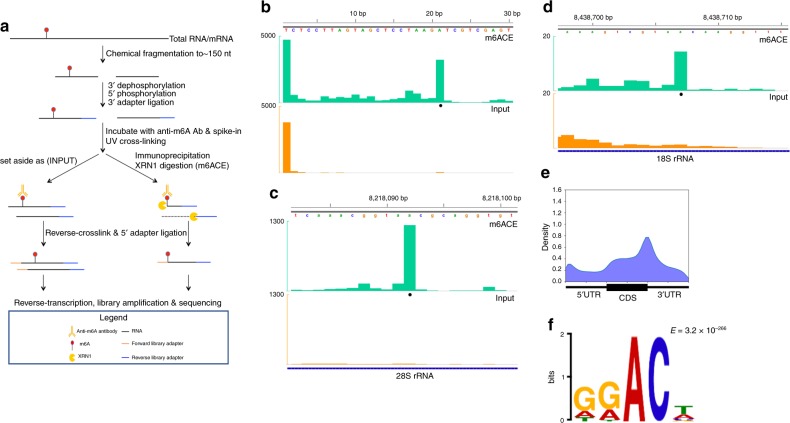
**m6ACE treatment enriches for RNA fragments with m6A in the first nucleotide.** **a** Procedure for m6ACE-seq. **b**–**d** m6ACE (green) and Input (orange) read-start counts (in reads per million mapped or RPM) mapped to either a synthetic RNA spike-in sequence (Supplementary Data 1) with a single m6A at position 21 (**b**), 28 S rRNA (**c**) or 18 S rRNA (**d**). Known m6A sites are denoted by black dots. Sequence corresponds to the same strand as the m6A site. Blue horizontal bars represent transcript models. **e** Metagene distribution profile of all significant m6A/m6Am sites in WT cells. **f** MEME analysis of the sequence context of all significant m6A/m6Am sites in WT cells.

Given the structural similarity between m6A RNA and N^6^-methyl-deoxyadenosine (6 mA) DNA, we capitalised on our recently reported single-base-resolution 6mA-sequencing method to develop m6ACE-seq^31^. Briefly, anti-m6A antibodies are first photo-crosslinked onto m6A-containing RNA, which are thus protected from subsequent 5′ to 3′ exoribonuclease treatment (Fig. 1a). Sequencing of protected RNA fragments should theoretically reveal high-resolution detection of m6A locations. We first tested m6ACE-seq on a synthesised RNA oligonucleotide containing a single m6A nucleotide at position 21 (Supplementary Data 1). Comparison of m6ACE reads to untreated input reads revealed a m6ACE-specific pileup of reads starting exactly at the m6A position (Fig. 1b). We also performed m6ACE-seq on RNA extracted from human HEK293T cells and focused on the 18S and 28S rRNA, which each have a single well-established m6A site^32^. Here, we observed the same m6ACE-specific pileup of read-starts at established 18S rRNA and 28S rRNA m6A positions (Fig. 1c, d). This demonstrates that exonucleases are able to digest away RNA up to and including the nucleotide just 5′ of the antibody-protected m6A nucleotide. Therefore, these m6ACE-seq profiles show that m6ACE treatment can be used to map exact locations of m6A within RNA.

### m6ACE-seq maps human m6A at single-base-resolution

We proceeded to explore the utility of m6ACE-seq to map m6A at a transcriptome-wide level by subjecting HEK293T polyA-selected RNA to m6ACE-seq. In order to assess the authenticity of m6ACE-seq identified m6A sites, we first focused on m6A sites that had previously been authenticated by an orthogonal sequencing-independent single-base-resolution m6A mapping technique known as SCARLET^33^. Across the long non-coding RNA *Malat1* and multiple mRNAs, m6ACE-seq read-starts exhibited sharp pileups at all 7 SCARLET-positive m6A sites and only 1 out of 6 SCARLET-negative sites (Supplementary Fig. 1a–d). This further supports the sensitivity and specificity of m6ACE-seq. We noticed that read-starts sometimes form a clustered noise pattern at locations without established m6A sites (Supplementary Fig. 1a). Close observation of SCARLET-positive sites also showed that besides the distinct pileup of read-starts at the exact site of m6A methylation, there is occasionally a mild pileup of read-starts at positions −4 to −3 of established m6A sites (Supplementary Fig. 1a). Given that we had also noticed similar trends for m6ACE-seq reads within our synthesised m6A RNA oligonucleotide, 18S rRNA and 28S rRNA (Fig. 1b–d), we factored these read patterns into our m6ACE-seq analysis for identifying m6A sites transcriptome-wide (see Methods). Consequently, m6ACE-seq identified 33,163 significant sites within the human transcriptome. Within mRNA, m6A is known to localise preferentially within the CDS and 3′UTR proximal to the stop codon^1,2^. As expected, metagene analysis of our identified m6A sites recapitulated this localisation (Fig. 1e). Consensus motif analysis of the sequences around all significant m6A sites also depicted a “DRm6ACH” motif, typical of human m6A sites (Fig. 1f)^17^. m6A sites identified by m6ACE-seq also exhibited significant overlap with sites identified by previous single-base-resolution m6A-sequencing methods (Supplementary Fig. 1e)^17,26^. We next tested the effect of using different m6A-specific antibodies on m6ACE and found that sites detected by other antibodies also exhibited significant overlap with those detected using the main m6A-specific antibody (Supplementary Fig. 1f; Supplementary Data 2 and 3). Amongst the sites that exhibited overlap between different sequencing methods or between different m6A-specific antibodies, these sites were methylated at a higher stoichiometric level as determined using an orthogonal approach (Supplementary Fig. 1g, h). This suggests that non-overlapping sites do not necessarily represent false-positive sites but might instead represent m6A sites with lower stoichiometric methylation that are more challenging to detect.

We noticed a m6A candidate site in the mitochondrial 16S rRNA that was not identified in previous single-base-resolution methylomes (Supplementary Fig. 1i). In order to validate this candidate site, we utilised a T3-DNA-ligase-based m6A detection assay, where DNA probes anneal to sequences flanking the queried site, and ligation efficiency of the probes is inversely correlated with methylation level at the site of query (Supplementary Fig. 1j)^34^. When compared to 2 separate 16S rRNA sites without strong m6ACE signals, ligation of probes flanking our candidate m6A site was ~29-fold less efficient, thereby validating it as a novel m6A site (Supplementary Fig. 1i, k). Together, these findings show that m6ACE-seq is capable of single-base-resolution transcriptome-wide mapping of established and novel m6A sites.

### m6ACE-seq quantitatively maps METTL3-dependent m6A

In the process of optimising m6ACE-seq, we spiked in synthesised methylated RNA into biological RNA samples to allow for normalisation of m6ACE efficiency across samples, as well as used sequencing adaptors with unique molecular identifiers (UMIs) to correct for any library amplification bias (Supplementary Data 1). The random UMI sequence at the 3′end of the 5′ adaptor, which ligates directly to m6A in the first position of the RNA fragment, also helped to minimise any ligation biases that might be caused by sequences directly downstream of m6A (Supplementary Fig. 2a–d)^35^. Using these normalisation and correction steps, we calculated the relative methylation levels (RML, see Methods) of individual m6A candidates between two triplicate sets of wild type (WT) samples. We observed strong correlation in the RMLs between the two sets, demonstrating high reproducibility of our method (Supplementary Fig. 2e).

**Fig. 2:**
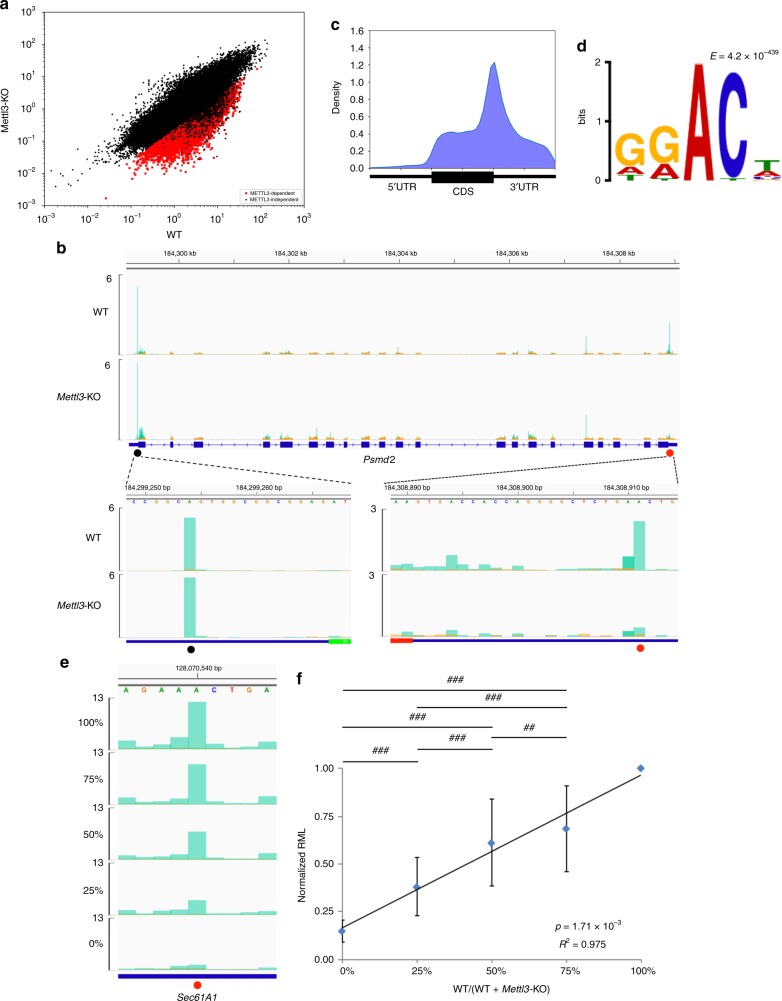
**m6ACE-seq quantitatively maps m6A reductions at individual METTL3-dependent m6A sites.** **a** Scatterplot of average RML of WT versus *Mettl3*-KO cells. METTL3-dependent are sites with RML reductions of at least log_2_fold of 2 (Student’s *t*-test *p* < 0.05) in *Mettl3*-KO compared to WT cells. **b**, **e** m6ACE (green) overlaid on Input (orange) read-start RPM counts mapped to a representative gene. METTL3-independent and dependent sites are respectively denoted by a black or red dot. Sequence corresponds to the same strand as the m6A site. Blue horizontal bars represent transcript models, with green and red sections respectively representing the start and stop codons. Magnified views of the 5′UTR and 3′UTR are also displayed for (**b**). For **e**, percentages represent proportion of WT RNA in a mixture of WT and *Mettl3*-KO RNA. **c** Metagene distribution profile of all METTL3-dependent m6A. **d** MEME analysis of the sequence context of METTL3-dependent m6A. **f** Plot of normalised RML against percentage of WT RNA in mixtures of WT and *Mettl3*-KO. RML of METTL3-dependent sites in each mixture was normalised to the corresponding RML in 100% WT RNA. Averages of triplicate normalised RML are represented with error bars (s.d.). A linear regression fit of the plot is depicted with its *R*^2^ value and *p*-value. ## and ### respectively denote *p* < 10^−145^ and *p* < 10^−307^ (Student’s *t*-test).

Subsequently, we hypothesised that m6ACE-seq should be capable of quantifying differential m6A methylation between different samples. To test this, we used CRISPR-Cas9 to knock out (KO) *Mettl3* to see if the isolated RNA would manifest METTL3-dependent m6A loss via m6ACE-seq (Supplementary Fig. 2f). In the absence of METTL3 expression, we observed 15,073 m6A sites exhibiting significant RML reductions (Fig. 2a; Supplementary Data 4). Close inspection of individual genes depicted a clear loss of m6ACE-seq read-start counts at “DRm6ACH” motifs in the 3′UTR proximal to the stop codon (Fig. 2b; Supplementary Fig. 2g). Metagene analysis of identified METTL3-dependent m6A sites exhibited a tighter localisation towards the stop codon than the collection of all METTL3-dependent and independent sites (Figs. 1e and 2c). Consensus motif analysis of METTL3-dependent m6A sites also depicted the “DRm6ACH” motif that is associated with METTL3-targeted m6A (Fig. 2d)^17^. Together, this validates the accuracy of m6ACE-seq.

It is known that for any given m6A site, only a fraction of harbouring transcripts are methylated^33^. To evaluate the potential of m6ACE-seq to quantify differential methylation fractions between samples, we mixed varying proportions of WT and *Mettl3-*KO RNA in order to simulate varying fractions of in vivo transcript methylation on a transcriptome-wide scale. As the fraction of WT RNA in the RNA mixture decreased, there was a corresponding decrease in m6ACE-seq read-start counts at individual m6A sites (Fig. 2e; Supplementary Fig. 2h). On a transcriptome-wide level, normalised RMLs were linearly correlated with the percentage of WT RNA present in the mixture (Fig. 2f; Supplementary Data 5). This demonstrates that m6ACE-seq is capable of quantifying differential methylation fractions of each m6A site across different samples.

### m6ACE-seq quantitatively maps PCIF1-dependent m6Am

We next sought to determine if m6ACE-seq can also map m6Am and focused on histone mRNAs that were previously shown to contain m6Am^36^. We observed clear m6ACE-seq read-start pileups at or near the first nucleotide of histone mRNAs, indicative of m6Am (Supplementary Fig. 3a, b). We also observed strong m6ACE-seq read-start signals within mRNA 5′UTRs that remained undiminished in *Mettl3*-KO cells (Fig. 2b; Supplementary Fig. 2g). Given their localisation near annotated transcription-start-sites (TSS) and their independence from *Mettl3*, these m6ACE-seq read-starts likely represent m6Am sites located in the first transcribed nucleotide. Together, these highlight an additional utility of m6ACE-seq in that it can identify both m6A and m6Am.

**Fig. 3:**
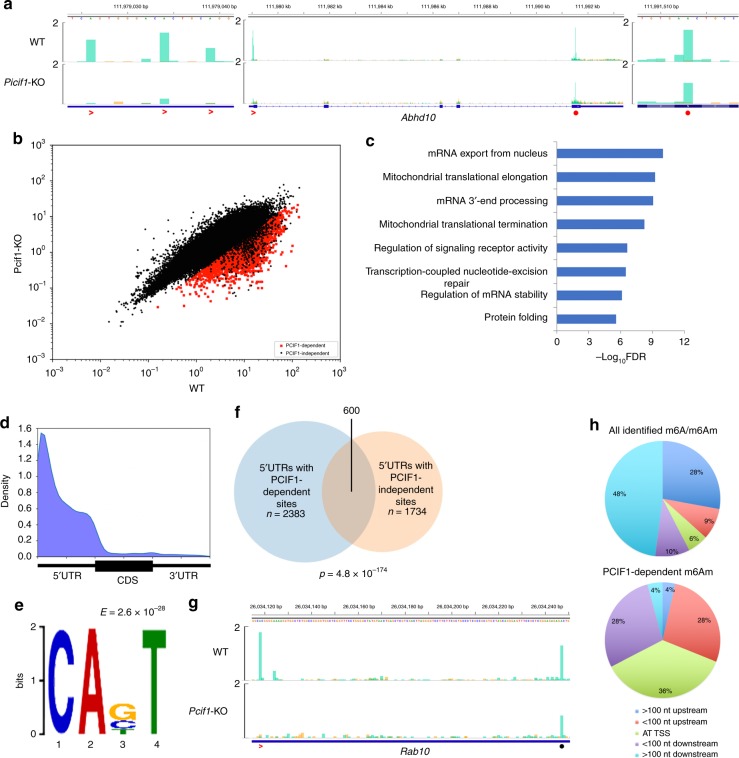
**m6ACE-seq quantitatively maps RML reductions at individual PCIF1-dependent m6Am sites.** **a** m6ACE (green) overlaid on Input (orange) read-start RPM counts mapped to a representative gene (center). PCIF1-dependent and METTL3-dependent sites are respectively denoted by red angles and red dots. Sequence corresponds to the same strand as the m6A/m6Am sites. Blue horizontal bars represent transcript models. Magnified views of the 5′UTR (left) and 3′UTR (right) are also displayed. **b** Scatterplot of average RML of WT versus *Pcif1*-KO cells. PCIF1-dependent are sites with RML reduction of at least log_2_fold of 2 (Student’s *t*-test *p* < 0.05) in *Pcif1*-KO compared to WT cells. **c** Functional annotation enrichment of genes containing PCIF1-dependent m6Am with significance of enrichment denoted as −log_10_FDR. **d** Metagene distribution profile of PCIF1-dependent m6Am. **e** MEME analysis of the sequence context of PCIF1-dependent m6Am. **f** Venn diagram representing overlap between 5′UTRs containing PCIF1-dependent m6Am with 5′UTRs containing PCIF1-independent sites. *p*-value of overlap by chance was calculated with a hypergeometric test. PCIF1-independent sites are sites where average RML reduction from WT to PCIF-KO is not significantly more than 1.5 fold (Student’s *t*-test *p* < 0.05). **g** m6ACE (green) overlaid on Input (orange) read-start RPM counts mapped to a representative 5′UTR. PCIF1-dependent and PCIF1-independent sites are respectively denoted by red angles and black dots. Sequence corresponds to the same strand as the m6A/m6Am sites. Blue horizontal bars represent transcript models. **h** Pie charts representing alignment of all identified m6A/m6Am (top) or PCIF1-dependent m6Am (bottom) with respect to CAGE-seq annotated TSSs^39^.

PCIF1 has a predicted N6-methyladenine methyltransferase domain and interacts with the phosphorylated C-terminal tail of RNA polymerase II during RNA transcription^37^. We subjected *Pcif1*-KO RNA to m6ACE-seq and found that PCIF1 depletion caused clear RML reductions in sites within 5′UTRs where m6Am resides (Fig. 3a, g; Supplementary Fig. 3a–f, h). If PCIF1 specifically catalyses m6Am methylation adjacent to mRNA caps, we would also expect METTL3-dependent sites proximal to stop codons to be resistant to PCIF1 depletion. We tested this by focusing on mRNAs with both *Pcif1*-KO-induced RML reductions in 5′UTRs and METTL3-dependent m6As in the associated 3′UTRs. Indeed, METTL3-dependent m6As in these mRNAs did not exhibit any RML reduction in PCIF1-KO cells (Fig. 3a; Supplementary Fig. 3f). We subsequently extended our analysis to identify all PCIF1-dependent sites, which amounted to 4264 throughout the transcriptome (Fig. 3b; Supplementary Data 6). These sites were found in genes enriched for functional annotations that focused on RNA processing and mitochondrial translation processes (Fig. 3c). Unlike METTL3-dependent m6A, PCIF1-dependent sites recapitulated a mRNA localisation shifted strongly towards the 5′UTR (Fig. 3d). PCIF1-dependent sites also exhibited a ‘CABU’ consensus motif that is a subset of the initiator consensus ‘BBCABW’ sequence found at TSSs, and is distinct from the ‘DRm6ACH’ motif of METTL3-dependent m6A (Fig. 3e; ‘A’ denotes the TSS, B = C/G/U, W = A/U)^38^. We also found no significant overlap in sites that are dependent on METTL3 versus PCIF1, further supporting PCIF1 as a methyltransferase that mediates methylation of sites distinct from that of METTL3 (Supplementary Fig. 3g). Together, these analyses validate the identity of these PCIF1-dependent sites as TSS-associated m6Am.

We next sought to determine what insights we can garner from utilising single-base-resolution m6ACE-seq to map m6Am, which we would otherwise miss out if we instead used a low-resolution m6A-sequencing technique. For example, 5′UTRs can harbour both PCIF1-independent m6A and PCIF1-dependent m6Am. In fact, we found that more than a quarter of 5′UTRs that contain PCIF1-dependent m6Am also contain PCIF1-independent m6A (Fig. 3f). Closer observation of some of these 5′UTRs revealed that PCIF1-independent m6A can be quite proximal to PCIF1-dependent m6Am (Fig. 3g; Supplementary Fig. 3h). In such cases, a low-resolution m6A-sequencing method might not be able to detect m6Am methylation loss after PCIF1-depletion simply because of the proximal m6A, resulting in false-negatives.

An alternative way to map m6Am might be to simply identify annotated TSSs within broad methylated regions identified by low-resolution m6A-sequencing. To test the plausibility of such a strategy, we first compared how well our identified PCIF1-dependent m6Am aligned with TSSs identified previously via cap-analysis-gene-expression-sequencing (CAGE-seq)^39^. Compared to all detected m6A/m6Am, PCIF1-dependent m6Am are indeed more enriched for TSS-alignment (Fig. 3h). However, there still exists a good proportion of m6Am sites that are slightly misaligned with respect to annotated TSSs, likely because of TSS heterogeneity (Fig. 3a, g; Supplementary Fig. 3b, d–f)^39^. This argues against the accuracy of using low-resolution m6A-sequencing to detect m6Am sites. Furthermore, almost 700 5′UTRs contain multiple PCIF1-dependent m6Am sites (Fig. 3a; Supplementary Fig. 3e, f), which is probably an underestimation as certain m6Am sites might be wrongly misannotated to be upstream of TSSs. Low-resolution m6A-sequencing will not be capable of detecting the individual m6Am sites in these 5′UTRs, leading to greater false-negative rates. Collectively, these findings demonstrate the importance of using single-base-resolution m6ACE-seq to accurately map m6Am.

### METTL16 depletion indirectly reduces global RNA methylation

**Fig. 4:**
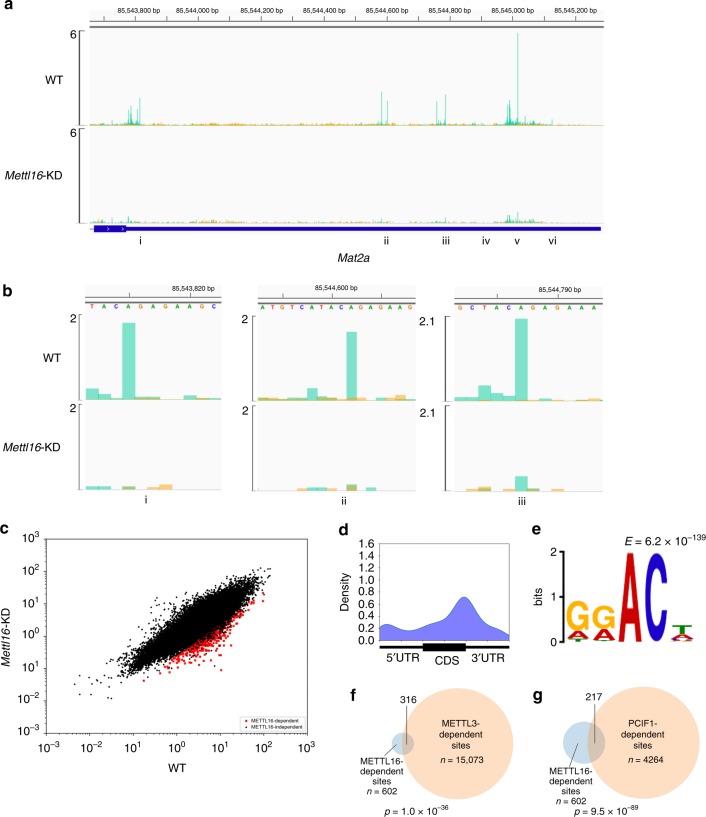
**METTL16 depletion reduces methylation of a plethora of m6A sites beyond its direct UACAGAGAA targets.** **a** m6ACE (green) overlaid on Input (orange) read-start RPM counts mapped to the Mat2a 3′UTR. Locations of ‘UACAGAGAA’ and similar motifs are denoted by roman numerals. Sequence corresponds to the same strand as the m6A site. Blue horizontal bars represent transcript models. **b** Magnified representations of the *Mat2a* 3′UTR at positions i, ii and iii as denoted in **a**. **c** Scatterplot of average RML of WT versus *Mettl16*-KD cells. METTL16-dependent are sites with RML reduction of at least log_2_fold of 2 (Student’s *t*-test *p* < 0.05) in *Mettl16*-KD compared to WT cells. **d** Metagene distribution profile of METTL16-dependent m6A/m6Am. **e** MEME analysis of the sequence context of METTL16-dependent m6A/m6Am. **f**, **g** Venn diagram representing overlap between METTL16-dependent with METTL3-dependent (**f**) or PCIF1-dependent (**g**) sites. *p*-value of overlap by chance was calculated with a hypergeometric test.

METTL16 is another m6A methyltransferase that mediates m6A methylation in the ‘UACm6AGAGAA’ motif^20^. *Mat2a* encodes a S-adenosyl-methionine (SAM) synthetase and its 3′UTR possesses 5 ‘UACAGAGAA’ sites. However, previous efforts to map m6A sites in this region with low-resolution m6A-RIP-seq yielded only 4 broad peaks dependent on METTL16^20^. To determine if m6ACE-seq could precisely map METTL16-dependent m6A sites, we knocked down (KD) *Mettl16* expression and extracted cellular RNA for m6ACE-seq (Supplementary Fig. 4a). Focusing first on the *Mat2a* transcript, we found clear *Mettl16*-KD-dependent reductions in read-start signals at all 5 ‘UACAGAGAA’ sites, and even at a similar ‘UACAGAAAA’ site within the *Mat2a* 3′UTR (Fig. 4a, b; Supplementary Fig. 4b). We also observed *Mettl16*-KD-dependent read-start signals reductions located exactly at a previously established METTL16-dependent m6A site within U6 snRNA (Supplementary Fig. 4c)^20^. The ability of m6ACE-seq to map m6A in various sequence contexts also demonstrates that m6ACE-seq does not exhibit sequence bias restrictions in mapping m6A.

We extended our analysis to identify other METTL16-dependent sites and found 602 throughout the transcriptome (Fig. 4c; Supplementary Data 7). We note that METTL16 was previously shown to induce splicing of the *Mat2a* transcript and expression of MAT2A protein to synthesise SAM^20^. As such, METTL16 depletion decreases MAT2A expression, resulting in reduced intracellular SAM, a key substrate required for RNA methylation. Therefore, loss of METTL16 can result in a transcriptome-wide loss of m6A methylation beyond sites directly methylated by METTL16. In support of this possibility, metagene analysis revealed that the identified METTL16-dependent m6A do not exhibit a localisation pattern that is unique from that of the collection of all m6A sites (Figs. 1e and 4d). Motif analysis also showed that sequences centred at METTL16-dependent m6A sites depicted the METTL3-dependent ‘DRm6ACH’ motif rather than the METTL16-dependent ‘UACm6AGAGAA’ (Fig. 4e). In accordance with this, more than half of METTL16-dependent m6A were co-identified as METTL3-dependent m6A (Fig. 4f). A substantial portion of METTL16-dependent m6A were also co-identified as PCIF1-dependent m6Am (Fig. 4g; Supplementary Fig. 4d). This suggests that METTL3 and PCIF1 are directly responsible for mediating methylation of identified METTL16-dependent sites. Together, these analyses argue that METTL16 mediates the methylation of the majority of METTL16-dependent m6A through an indirect mechanism, likely via controlling intracellular SAM levels.

### ALKBH5 suppresses accumulation of m6A

**Fig. 5:**
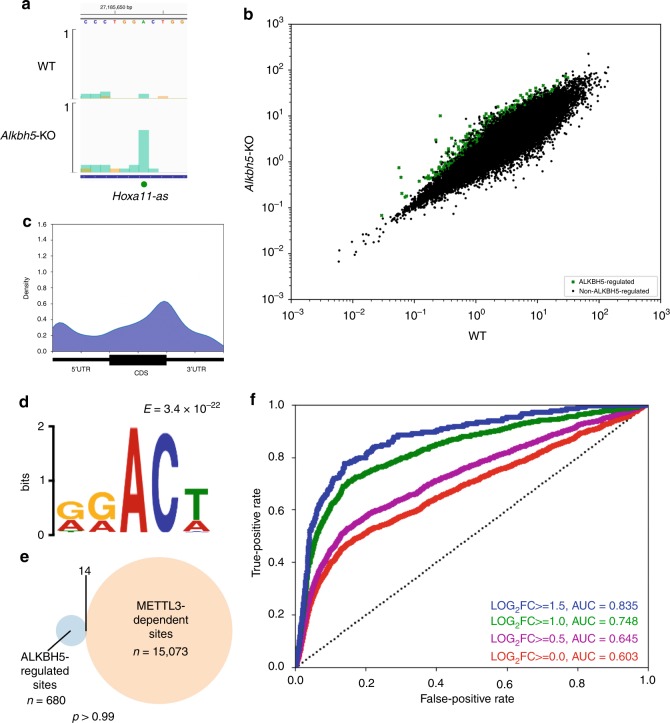
**ALKBH5 suppresses accumulation of m6A.** **a** m6ACE (green) overlaid on Input (orange) read-start RPM counts mapped to a representative gene. ALKBH5-regulated sites are denoted by green dots. Sequence corresponds to the same strand as the m6A sites. Blue horizontal bars represent transcript models. **b** Scatterplot of average RML of WT versus *Alkbh5*-KO cells. ALKBH5-regulated are sites with RML accumulation of at least log_2_fold of 1 (Student’s *t*-test *p* < 0.05) in *Alkbh5*-KO compared to WT cells. **c** Metagene distribution profile of ALKBH5-regulated sites. **d** MEME analysis of the sequence contexts of ALKBH5-regulated sites. **e** Venn diagrams representing overlaps between ALKBH5-regulated sites with METTL3-dependent m6A. *p*-value of overlap by chance was calculated with a hypergeometric test. **f** Receiver operator characteristic (ROC) curve for how well ALKBH5-regulated sites are at predicting steady-state non-methylated sites in WT cells. Each curve represents ALKBH5-regulated sites as defined by a different minimum log_2_FC RML accumulation cutoff (Student’s *t*-test *p* < 0.05) in *Alkbh5-*KO over WT cells. Area-under-curve (AUC) values for each log_2_FC are also provided.

While m6ACE-seq is able to quantitatively map methyltransferase-dependent m6A/m6Am, we wanted to test if m6ACE-seq can also quantify loss of demethylation in demethylase-depleted cells. ALKBH5 is a *Alkb* family iron(II)/alpha-ketoglutarate-dependent dioxygenase that has a strong capacity to demethylate m6A^22^. Therefore, we depleted ALKBH5 and used m6ACE-seq to identify m6A with RML accumulation in *Alkbh5*-KO cells (Supplementary Fig. 5a; Supplementary Data 8). As expected, we observed RML accumulation in individual ‘DRm6ACH’ sites after ALKBH5-depletion (Fig. 5a; Supplementary Fig. 5b, c). On a transcriptome-wide scale, we found 680 sites with RML accumulations after ALKBH5-depletion (Fig. 5b). ALKBH5-regulated sites were found to localise in a manner similar to stop-codon proximal METTL3-dependent m6A (Figs. 2c and 5c). Motif analysis revealed that ALKBH5-regulated sites also exhibited a consensus motif similar to the METTL3-dependent ‘DRm6ACH’ (Fig. 5d).

We subsequently enquired what the purpose of demethylating these ALKBH5-regulated sites is and envisioned two possibilities. First, these are dynamic sites that are initially methylated in the nucleus to allow for RNA-methylation-mediated regulation before being demethylated by ALKBH5 in the cytoplasm after said regulation has occurred (Supplementary Fig. 5d). Second, methylation on these sites is to be avoided and thus, ALKBH5 acts to suppress these undesired modifications while the RNA is still in the nucleus so as to maintain them in a constantly unmethylated state (Supplementary Fig. 5e). We reckoned that we could distinguish between the two possibilities by comparing the METTL3-dependent methylome with the ALKBH5-regulated methylome. Specifically, if ALKBH5-regulated sites are dynamic, they should exhibit a dynamic RML range that not only exhibits accumulation in ALKBH5-KO cells but also reduction in METTL3-depleted cells, allowing them to be co-identified as METTL3-dependent sites too (Supplementary Fig. 5d). However, this is not the case as hardly any ALKBH5-regulated sites were co-identified as METTL3-dependent sites (Fig. 5e). We further reasoned that if ALKBH5-regulated sites are dynamic, they should on average exhibit a significant level of steady-state methylation in WT cells (Supplementary Fig. 5d). To test this, we calculated the receiver-operator-characteristics-area-under-curve (ROCAUC) for how well ALKBH5-regulated sites were at predicting methylation absences at those very sites in WT cells. This revealed that the stronger a site is regulated by ALKBH5, the higher its likelihood of being unmethylated at steady-state in WT cells (Fig. 5f). If not for their RML accumulations in *Alkbh5*-KO cells, the majority of ALKBH5-regulated sites would not even be identified to have the capacity to be methylated in WT cells. These findings argue against a model where ALKBH5-regulated sites are dynamic and instead suggest that ALKBH5 continuously acts on its regulated sites to keep them constantly and completely unmethylated in WT conditions (Supplementary Fig. 5e).

### FTO loss causes disruptive m6Am accumulation

**Fig. 6:**
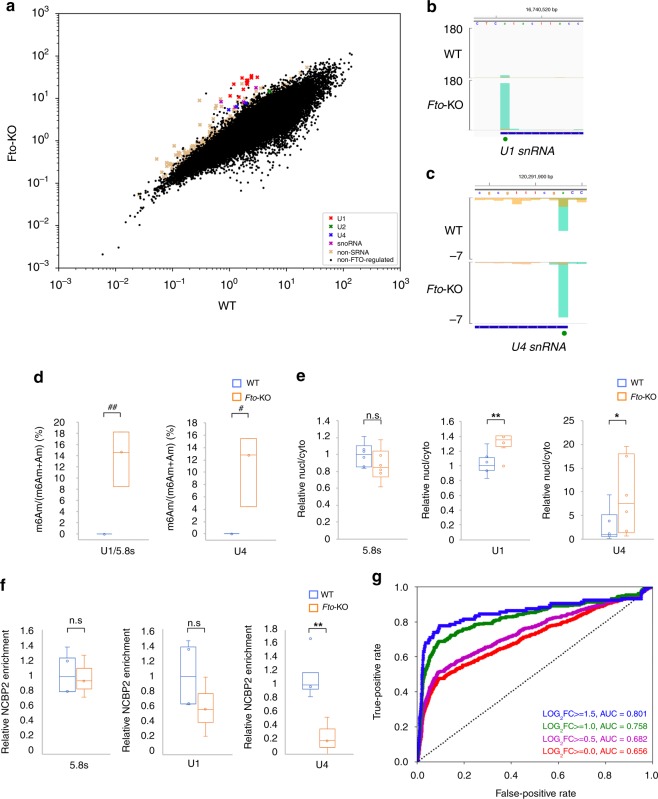
**FTO loss causes m6Am accumulation that disrupts binding of specific snRNA precursors to nuclear export machinery.** **a** Scatterplot of average RML of WT versus *Fto*-KO cells. FTO-regulated (‘X’s) are sites with RML accumulation of at least log_2_fold of 1 (Student’s *t*-test *p* < 0.05) in *Fto*-KO compared to WT cells. **b**, **c** m6ACE (green) overlaid on Input (orange) read-start RPM counts mapped to the 5′ ends of *U1 snRNA* (**b**) and *U4 snRNA* (**c**). FTO-regulated m6Am sites are denoted by green dots. Sequence corresponds to the same strand as the m6A/m6Am sites. Blue horizontal bars represent transcript models. **d** Box and whisker plots of m6Am/(m6Am + Am) percentages in WT (blue) and *Fto*-KO (orange) cells as measured by UHPLC-MS/MS. Represented are median and interquartile ranges for 3 biological replicates. # and ## denote *p* < 0.05 and 0.005 respectively (Student’s *t*-test). Values for WT cells are in the non-quantifiable range. **e** Box and whisker plots of ratio of sRNA levels (relative to *7SL scRNA*) in nucleus over cytoplasm in WT (blue) and *Fto*-KO (orange) cells. Represented are median and interquartile ranges for 6 biological replicates, normalised to the corresponding median values in WT cells. A higher ratio corresponds to the sRNA localising more to the nucleus than the cytoplasm. **f** NCBP2-RIP analysis of WT (blue) and *Fto*-KO (orange) lysates. Relative NCBP2 enrichment is the ratio of each sRNA pulled down with anti-NCBP2 over non-specific IgG, normalised to housekeeping *Gapdh* mRNA. Represented are box and whisker plots with median and interquartile ranges for at least three biological replicates, normalised to the corresponding median values in WT cells. **g** ROC curve for FTO regulation as a predictor of steady-state non-methylation in WT cells. Each curve represents FTO-regulated sites as defined by a different minimum log_2_FC RML accumulation cutoff (Student’s *t*-test *p* < 0.05) in *Fto-*KO over WT cells. AUC values for each log_2_FC are also provided. For box and whisker plots, centre line, box boundaries, lower whisker, upper whisker and empty circles represent the median, interquartile range, minimum value, maximum value and outliers respectively. n.s., * and ** respectively denote not significant, *p* < 0.1 and *p* < 0.01 (Student’s *t*-test).

FTO has also been shown to possess both m6A and m6Am demethylation activity in vitro but recent studies have reported conflicting results about its in vivo target^9,23–29^. To resolve this, we performed m6ACE-seq on *Fto*-KO RNA and identified 273 sites with RML accumulations as FTO-regulated sites (Fig. 6a; Supplementary Fig. 6a; Supplementary Data 9). Amongst these sites, we were able to find evidences of FTO-depletion induced RML accumulation at ‘DRACH’ sites within CDSs and 3′UTRs, typical of METTL3-dependent ‘DRm6ACH’ (Supplementary Fig. 6b, c). Compared to mRNAs, transcripts that exhibited the greatest RML accumulations in FTO-KO cells were the small RNAs (sRNAs), specifically small nucleolar RNAs (snoRNA) and small nuclear RNA (snRNA) (Fig. 6a). Upon close inspection of these sRNAs, we observed that the exact site of significant RML accumulation generally matched the first nucleotide of each sRNA (Fig. 6b, c; Supplementary Fig. 6d). The first nucleotide of Sm-class snRNAs are also known to be methylated at the 2′-O-ribose^40^. Therefore, the RML accumulation at the first nucleotide of snRNAs ought to represent reduced *N*^6^-demethylation of m6Am. To test this, we used ultra-high-performance-liquid-chromatography coupled with tandem mass spectrometry (UHPLC-MS/MS) to quantify Am and m6Am levels, focusing on U1 and U4 snRNAs as these snRNAs exhibited robust RML accumulations upon *Fto*-KO (Fig. 6a–c). While there was no quantifiable snRNA m6Am in WT cells, there were indeed significant m6Am levels in both snRNAs in *Fto*-KO cells (Fig. 6d; Supplementary Fig. 6e–g).

We next sought to characterise the functional consequence of losing FTO-directed RNA demethylation by checking if m6Am accumulation in U1 and U4 snRNAs affected their RNA levels in comparison to 5.8 s rRNA, a sRNA without m6Am (Supplementary Fig. 6h). We found that *Fto*-KO did not result in any significant changes in the relative RNA levels of any snRNA (Supplementary Fig. 6i). Since the maturation of Sm-class snRNAs involves translocation between the nucleus and cytoplasm, we isolated nuclear and cytoplasmic fractions and quantified if any of the Sm-class snRNAs displayed a shift in their cellular localisations (Supplementary Fig. 6j)^41^. We found that U1 and to a greater degree, U4 snRNAs were more confined to the nucleus in *Fto*-KO cells (Fig. 6e). U2 and U5 snRNAs also exhibited RML accumulations in the first nucleotide in the absence of FTO but the accumulations were neither as significant nor as robust as those of U1 and U4 snRNAs (Fig. 6a). Likewise, U2 and U5 snRNAs both did not exhibit any shifts in cellular localisation similar to that of U1 and U4 snRNAs (Supplementary Fig. 6k).

The increased nuclear localisation of specific snRNAs could be caused either by inhibition of nuclear export of the initial precursor snRNA or improved nuclear import of the mature snRNA^41^. Sm-class snRNA precursors possess 5′ caps that bind to NCBP2 within the cap-binding complex, which mediates export of bound capped transcripts out of the nucleus, so we tested if m6Am accumulation affects snRNA binding to NCBP2. Using NCBP2 RNA immunoprecipitation (RIP; Supplementary Fig. 6l), we found that NCBP2 binding to *Fto*-KO U4 snRNA was ~6-fold weaker than to WT U4 snRNA (Fig. 6f). Therefore, in the absence of FTO, m6Am methylation accumulation in the first nucleotide of U4 snRNA disrupts binding of the adjacent snRNA cap to NCBP2. This results in decreased nuclear export of U4 snRNA precursor and increased nuclear retention.

We next enquired if FTO suppresses methylation of its regulated sites (Supplementary Fig. 5e) or if it mediates cytoplasmic methylation-reversal as previously reported (Supplementary Fig. 5d)^25^. Similar to ALKBH5-regulated sites, hardly any FTO-regulated sites were co-identified as methyltransferase-dependent sites (Supplementary Fig. 6m, n). Furthermore, ROCAUC analysis revealed that the degree of FTO-mediated demethylation is strongly predictive of a FTO-regulated site being unmethylated in WT cells at steady state (Fig. 6g). Therefore, FTO acts to keep its regulated sites constantly and completely unmethylated.

### FTO overexpression suppresses nuclear RNA methylation

**Fig. 7:**
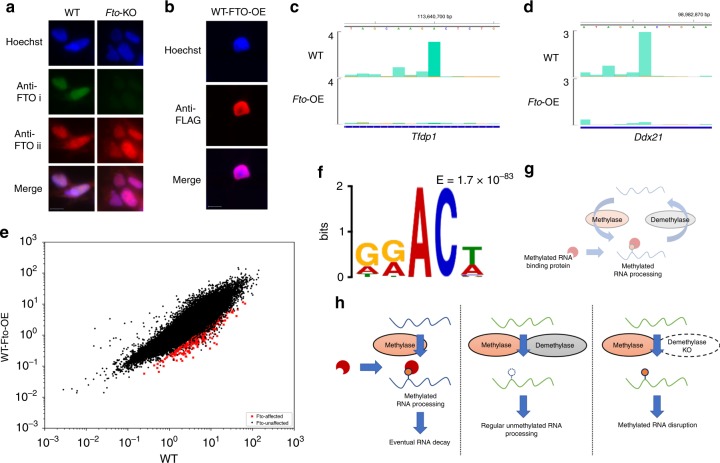
**FTO overexpression causes aberrant mRNA methylation-suppression in the nucleus.** **a** Immunofluorescence images of WT versus *Fto*-KO cells. Anti-FTO i and anti-FTO ii antibodies are respectively Abcam ab126605 and Santa Cruz sc271713. Anti-FTO i is specific for endogenous FTO while anti-FTO ii is not. Scale bar denotes 10 µm. **b** Immunofluorescence images of C-terminal-3 × -FLAG-tagged-WT-FTO in HEK293T. Scale bar denotes 10 µm. **c**, **d** m6ACE (green) overlaid on Input (orange) read-start RPM counts mapped to representative mRNAs. Sequence corresponds to the same strand as the m6A site. Blue horizontal bars represent transcript models. **e** Scatterplot of average RML of WT versus WT-*Fto*-OE cells. WT-FTO-affected are sites with RML reduction of at least log_2_fold of 2 (Student’s *t*-test *p* < 0.05) in WT-*Fto*-OE compared to WT cells. **f** MEME analysis of the sequence contexts of FTO-affected sites. **g** Model for cytoplasmic RNA methylation-reversal. **h** Model for disruptive methylation suppression by RNA demethylases. (Left panel) Most methylated RNAs (blue) are not demethylated by demethylases but simply undergo eventual RNA decay. (Middle panel) Selected RNAs (green) that ought to remain unmethylated are acted on almost simultaneously by both methyltransferase and demethylase, resulting in no net accumulation of RNA methylation. (Right panel) In the absence of demethylases, RNA methylation accumulates anomalously, which disrupts regular RNA processing.

FTO is mainly localised to the nucleus^21^. However, recent work reported the detection of cytoplasmic FTO, arguing that FTO can thus mediate RNA methylation-reversal in the cytoplasm^25,42^. We sought to validate FTO’s cellular localisation using a specific antibody and found FTO to be strictly nuclear-localised (Fig. 7a; anti-FTO i). Furthermore, we found that another antibody previously used to demonstrate FTO’s cytoplasmic localisation by immunofluorescence was actually non-specific (Fig. 7a; anti-FTO ii)^42^. This highlights the absolute necessity of using *Fto*-KO cells to validate FTO’s cellular localisation.

We next tested if WT-FTO overexpression (OE) caused any aberrant RNA demethylation. Through m6ACE-seq of FTO-OE RNA, we found that FTO overexpression indeed caused m6A reductions at 373 sites transcriptome-wide (Fig. 7b–e; Supplementary Data 10). These sites tend to exhibit a ‘DRm6ACW’ motif (Fig. 7f). Notably, none of the sites that exhibit RML accumulation in *Fto*-KO cells were affected by FTO overexpression. This was expected given that endogenous-FTO-regulated sites generally have no steady-state methylation in WT cells and thus cannot be aberrantly demethylated by exogenous FTO (Fig. 6g). Since the overexpressed FTO is strictly nuclear, the demethylation observed is likely overexpressed FTO aberrantly suppressing m6A methylation of mRNA before it shuttles out of the nucleus (Fig. 7b).

## Discussion

By photo-crosslinking specific antibodies, we were able to induce a tight protection of m6A/m6Am and its downstream RNA from 5′ to 3′ exoribonucleases. Sequencing of this protected RNA fragment as read-start pileups allowed us to map RNA methylation at single-base-resolution. We confirmed this property by precisely mapping known m6A locations in synthesised RNA oligonucleotides and m6A sites in multiple human RNAs that were previously established using an orthogonal approach^33^. We also demonstrated that m6ACE-seq is capable of mapping m6Am. Compared to CLIP-based m6A-sequencing methods, m6ACE-seq does away with inconvenient radioactive gel electrophoresis steps and effectively halves the time needed for library preparation^17,26,30^. Since m6ACE-seq maps methylation based on the pileup of read-starts as opposed to mutational signatures, m6A-mapping is also less sensitive to small nucleotide polymorphisms or random sequencing errors. The 5′ adaptor that we used to directly ligate to the methylated RNA terminates with a 3′ 8-mer UMI. Besides allowing us to eventually correct for amplification bias, this randomised UMI also limits any ligation bias that might wrongly bias certain RNA sequences to be mapped as methylated. For identifying m6A or m6Am, we normalised m6ACE-induced read-start pileups against random-fragmentation-induced read-start pileups in an input library constructed in parallel using the same RNA source with the exact same library construction steps except without immunoprecipitation and exoribonuclease treatment (Fig. 1a). This normalisation strategy is also used for m6A-RIP-seq but is generally absent from CLIP-based m6A-sequencing methods and analyses. Finally, we integrated methylated RNA spike-ins to correct for differences in crosslinking/immunoprecipitation/exonuclease efficiencies between samples. Overall, these steps allow m6ACE-seq to quantify differential methylation levels across sample types. For our own study, we used slightly relaxed significance cutoffs to generate more inclusive methylomes but we envision that future users can implement more stringent cutoffs for their m6ACE-seq analyses to generate higher-confidence methylomes that suit their experimental needs.

Using m6ACE-seq on cells individually depleted of every known methyltransferase or demethylase, we generated the first comprehensive atlas of single-base-resolution methylomes unique to each individual m6A/m6Am methyltransferase or demethylase. This atlas afforded us the ability to compare methylomes specific to distinct methyltransferases, and has helped to tackle previously unanswered questions. For example, despite exhibiting a strong specificity to methylate only ‘UACAGAGAA’ motifs, a previous report had used low-resolution m6A-RIP-seq and found that a majority of METTL16-dependent methylated regions lack any ‘UACAGAGAA’^20^. METTL16 was speculated to either directly methylate these regions through an unknown co-factor that directs METTL16 to non-‘UACAGAGAA’ sequences, or indirectly through modulating intracellular SAM levels, which is a substrate required for RNA methylation. Comparison of METTL3- and PCIF1-dependent methylomes with METTL16-dependent methylomes at single-base-resolution allowed us to conclude in support of the latter indirect mechanism. METTL16’s ability to modulate the methylation and consequently, the processing of a plethora of m6A sites beyond its direct targets is perhaps a contributing reason to why it is an essential gene in mammalian cells^20^. We also focused on PCIF1, which was validated as a m6Am methyltransferase during the preparation of our manuscript^43,44^. Our single-base-resolution methylome of PCIF1-dependent m6Am sites highlighted that TSS heterogeneity, m6Am clustering or proximal PCIF1-independent sites can easily result in false-negatives. Similar to a recent study, our results here emphasise the need for pinpoint precision when mapping m6Am^45^. We envision that it is also plausible to use m6ACE-seq to map PCIF1-dependent m6Am maps as an alternative to CAGE-seq for TSS mapping.

Finally, our work also addresses an ongoing debate about whether the existence of m6A demethylases means that m6A and m6Am are reversible modifications^23,24,26,27^. Such a model purports that after RNA accumulates functional levels of methylation via methyltransferases in the nucleus, it can undergo methylation-mediated regulation before being demethylated in the cytoplasm, allowing the newly-unmethylated RNA to again function in the absence of RNA methylation (Fig. 7g). Based on analysis of our methylome atlas, we instead propose the following (Fig. 7h): Most RNAs that are methylated do not undergo demethylation and remain methylated until they decay in the cytoplasm. However, methylation of selected RNA sites can disrupt downstream RNA processing pathways and thus, these RNA sites are supposed to remain unmethylated. Despite this, these selected RNA sites are still targeted by methyltransferases, perhaps because they fulfil the consensus target motif. As such, demethylases actively and specifically demethylate these RNA sites while they are in the nucleus so as to suppress disruptive methylation from ever accumulating. Failure of demethylases to do this subjects these RNAs to unwanted regulatory pathways, which can have broad implications on cellular processes. Notably, we found that FTO-depletion causes more robust RNA methylation accumulation in snRNAs and snoRNAs than in mRNA, which was also reported by another recent study^28^. Our study further demonstrates that snRNAs have no quantifiable methylation in WT cells and only accumulates m6Am at the first nucleotide in the absence of FTO, indicating that FTO acts to suppress rather than reverse m6Am methylation. The resultant anomalous m6Am disrupts binding of snRNA precursors to nuclear export machinery, potentially impeding snRNA maturation and pre-mRNA splicing^41^. This likely contributes to the aberrant widespread exon exclusion phenotype previously observed in *Fto*-KO cells^46^. It is also noteworthy that out of all Sm-class snRNAs, the 5′ terminal of U4 snRNA best fits the PCIF1-dependent ‘Cm6AmBU’ consensus motif. Therefore, U4 snRNA is likely to be the snRNA most targeted by PCIF1, which explains why loss of demethylation by FTO affects U4 snRNA more severely than other Sm-class snRNAs.

Our data do not discount the possibility that m6A and m6Am might be dynamically reversed in response to certain cellular stresses or developmental triggers that cause functional ALKBH5 or FTO to translocate into the cytoplasm. Should such conditions ever be identified, use of m6ACE-seq will certainly help to validate any reversal of RNA methylation. How demethylases are directed to suppress methylation from accumulating on specific RNA targets and if this specificity changes in different tissues also remain to be determined. It is plausible that RNA secondary structure or RNA binding proteins that recruit RNA demethylases might contribute to the aforementioned specificity. We envision that an atlas of single-base-resolution methylomes in different cell types will help elucidate demethylase specificity mechanisms and identify new forms of epitranscriptomics-mediated RNA metabolism.

## Methods

### Tissue culture

ATCC HEK293T CRL-3216 cells were cultivated in a sterile 5% CO_2_ incubator at 37 °C, and in DMEM supplemented with 10% FBS and 1% penicillin/streptomycin. Cells within passage 3–20 were used for experiments. HEK293T cells were regularly subjected to MycoAlert Plus Mycoplasma kit (Lonza LT07) to verify that they were mycoplasma-free.

### siRNA Knockdown

HEK293T cells were first seeded in a 6-well plate with a seeding density of 4.8 × 10^5^ cells per well. After 24 h, HEK293T cells were then transfected with 22 nM (50pmoles per well) final concentration of respective siRNAs using RNAiMax transfection reagent (Invitrogen 13778) according to the manufacturer’s instructions. siRNA s35507 (Thermo Scientific) was used to knockdown *Mettl16*. Twenty-four hours post-transfection, HEK293T cells were diluted to new plates at a seeding ratio of 1:9 to allow the cells to divide for an additional 48 h before being harvested for RNA or protein (total 72 h knockdown).

### Generation of knockout cell lines using CRISPR-cas9

HEK293T gene deletions were performed as described^31^. Briefly, guide RNA sequences corresponding to a region around the start codon of the gene of interest were designed using CRISPOR, then cloned into pSpCas9 BB-2A-puro (Addgene 62988) plasmids. Pairs of guide RNAs (Supplementary Data 1) were designed to induce either frameshift mutation close to the 5′ end of the gene or to delete the start codon. HEK293T cells were plated in 12-well plates at 2 × 10^5^ cells per well in regular growth media but without antibiotics. 16–24 h later, cells were transfected with 500 ng of each of the pair of guide RNA-expressing plasmid via Lipofectamine 2000 (Thermofisher 11668). Twenty-four hours post-transfection, successfully transfected cells were selected via survival under a 72-h treatment with 2 µg ml^−1^ puromycin. Puromycin-resistant cells were expanded for monoclonal dilution to select for monoclones that present desired gene deletions. The knockout mutations in these monoclones were further verified by loss of protein of interest via Western blotting.

### Generation of overexpression cell lines

Overexpression cell lines were generated by transfecting HEK293T cells with plasmids containing FL-FTO inserted upstream of a 3X FLAG-tag. Cells were passaged after 24 h and expanded for an additional 48 h for RNA extraction or immunofluorescence.

### Cellular fractionation

Nuclear and cytoplasmic fractionation was performed using the Nuclei EZ prep nuclei isolation kit (Sigma NUC101) according to manufacturer’s instructions. Purified intact nuclei and cytoplasmic lysates were subjected to either RNA or protein isolation.

### RNA immunoprecipitation

~2 × 10^7^ cells were harvested and washed with cold PBS twice and lysed in 1 ml RIP-lysis buffer [200 mM NaCl, 50 mM Tris pH 8, 1 mM EDTA, 0.5% IGEPAL CA-630 (Sigma I8896), 100 U ml^−1^ SUPERase-in RNase inhibitor (Invitrogen AM2694), 1X Complete Mini EDTA-free protease inhibitor (Sigma 4693159001), 1X Phosphatase inhibitor cocktail 2 (Sigma P5726)] for 30 min on ice, with 10 s vortex-mixing every 10 min. Lysed cells were centrifuged at 16,000 × *g* at 4 °C for 30 min and the supernatant was collected as cell lysate. 1 mg of cell lysate was then mixed with 10 µg rabbit anti-NCBP2 antibody (Abcam ab91560) or 10 µg of rabbit IgG (Abcam ab37415) as a mock-RIP. Antibody-lysate mixture was tumbled at 4 °C for 2 h. 50 µl Dynabead-proteinA (Life Technologies 10002D) was washed six times with 1 ml cold RIP-wash buffer [150 mM NaCl, 10 mM Tris pH 8, 1 mM EDTA, 0.1% IGEPAL CA-630, 1X Complete Mini EDTA-free protease inhibitor] and mixed with the antibody-lysate mixture before being mixed by tumbling at 4 °C for 2 h. Beads were washed four times with 0.5 ml cold RIP-wash buffer with a single change of tube. Beads were either boiled in 40 µl 1X Laemmli buffer for western blotting or incubated in Trizol-LS (Ambion 10296) for RNA isolation.

### RNA isolation

Total RNA was isolated from adherent HEK293T using Trizol-LS (Ambion 10296) according to manufacturer’s instructions and quantified using the Qubit RNA HS assay (ThermoFisher Q32855).

### m6ACE library preparation

Total RNA was first ethanol precipitated again to remove residual salt. Poly(A) RNA was purified using Poly(A)Purist Mag kit (Thermofisher AM1922) according to manufacturer’s instructions. After another round of ethanol-precipitation, poly(A) RNA was fragmented to 120–150 nt by incubating in RNA fragmentation buffer (Ambion AM8740) for 7.5 min at 70 °C. Fragmented RNA was ethanol-precipitated then treated with 10U T4 PNK (NEB M0201) for 30 min at 37 °C before adding 1 mM ATP and incubating for an additional 30 min at 37 °C before purifying the RNA using Oligo Clean & Concentrator (Zymo D4060). 3′ ligation was then performed as described^47,48^, where 200 pmol 5′-adenylated,3-dideoxyC DNA adaptors were ligated with 400U truncated T4 RNA ligase 2 (NEB M0242) in 1X ATP-free T4 RNA ligase buffer [50 mM Tris pH 7.5, 60 µg ml^−1^ BSA, 10 mM MgCl_2_, 10 mM DTT, 12.5% PEG8000] for 2 h at 25 °C. Ligated RNA was purified with Ampure XP beads (Beckman Coulter A63881). 200 pg of 3′-ligated methylated RNA spike-in (Supplementary Data 1) was added to 1–5 µg of ligated Poly(A) RNA and the mixture was denatured for 5 min at 65 °C before incubating for 2 min on ice. This denatured RNA mixture was incubated overnight at 4 °C with 8 µg anti-m6A antibody (Synaptic Systems 202003 for all experiments except Synaptic Systems 202111 and Abcam 151230 for specific experiments) in 1X IP buffer [150 mM LiCl, 10 mM Tris pH 7.4, 0.1% IGEPAL CA-630 (Sigma I8896)] supplemented with 1U µl^−1^ RNasin Plus (Promega N2611). In parallel, 1.2 mg Dynabeads-Protein-A was blocked overnight at 4 °C in 1X IP buffer supplemented with 0.5 mg ml^−1^ BSA (Sigma A7906). The antibody-RNA mixture was split into 50 µl aliquots on ice and crosslinked with 0.15 J cm^−2^ 254 nm UV radiation six times. The antibody-RNA mixture was recombined and 1% of it was set aside as input-RNA and the remainder (designated as m6ACE-RNA) was mixed with decanted BSA-blocked Dynabeads-Protein-A for 1.5 hr at 4 °C. Beads bound with crosslinked RNA were then washed with 250 µl of the following cold buffers in this order: Wash buffer 1 [1 M NaCl, 50 mM HEPES-KOH pH 7.4, 1% Triton X-100, 0.1% Sodium Deoxycholate, 2 mM EDTA], Wash buffer 2 [0.5 M NaCl, 50 mM HEPES-KOH pH 7.4, 1% IGEPAL, 0.1% Sodium Deoxycholate, 2 mM EDTA], Wash buffer 3 [1% Sodium Deoxycholate, 25 mM LiCl, 10 mM Tris pH 8, 1% Triton X-100, 2 mM EDTA], TE [10 mM Tris pH 8, 1 mM EDTA] and finally 10 mM Tris pH 8. m6ACE RNA was then denatured in 10 µl 10 mM Tris pH 8 at for 5 min at 65 °C and for 2 min on ice. m6ACE RNA was subjected to 5′ to 3′ exonuclease treatment with 1U XRN-1 (NEB M0338) in XRN-1 buffer [100 mM LiCl, 45 mM Tris pH 8, 10 mM MgCl_2_, 1 mM DTT] and 1U µl^−1^ RNasin Plus shaking at 1krpm for 1 hr at 37 °C. The m6ACE RNA-bead mixture was then washed with Wash buffer 1, Wash buffer 2, Wash buffer 3, TE and 10 mM Tris pH 8. Both input and m6ACE RNAs were eluted in elution buffer [1%SDS, 200 mM NaCl, 25 mM Tris pH 8, 2 mM EDTA, 1 mg ml^−1^ Proteinase K (Thermo Scientific EO0491)], shaking at 1krpm for 1.5 hr at 50 °C. RNAs were ethanol-precipitated and ligated to 5pmol 5′adaptors (Supplementary Data 1) with 10U T4 RNA ligase (Ambion AM2140) supplemented with 12.5% PEG8000 and 2U µl^−1^ RNasin Plus for 16 hr at 16 °C before being purified with Oligo Clean & Concentrator. 5pmol of reverse transcription primer (Supplementary Data 1) was annealed (72 °C 2 min, ice 2 min) and reverse transcription was performed with 200U SuperscriptIII (Invitrogen 18080) for 1 hr at 50 °C, with the reaction stopped by incubating for 15 min at 70 °C. The cDNA was PCR amplified for 14 cycles with Phusion High-fidelity PCR mastermix (Thermo Scientific F530) and Truseq PCR primers. Finally, primer-dimer and adaptor-dimers were removed with Ampure XP beads before undergoing PE75 sequencing on the Illumina Nextseq platform.

### m6ACE-sequencing analysis

Fastq sequences were first trimmed of 5′ and 3′ adaptor sequences and poly(A) tails using Cutadapt^49^. The 8-mer ‘N7B’ (N = A/C/G/T, B = C/G/T) UMI located at the first 8 nucleotides of read 1 was registered and trimmed. Any complementary UMI sequence in read 2 was also trimmed. Reads were mapped to the methylated spike-in (Supplementary Data 1) using Bowtie2, or to the hg38 assembly transcriptome (Gencode v28 comprehensive gene annotations) using STAR^50,51^. Aligned pairs that had the same mapping coordinates and UMIs were filtered out as PCR duplicates. Read-start coordinates in hg38-mapped reads that began with an adenosine nucleotide, and had a minimum mean read count of 1 across the triplicate samples were collated. m6A or m6Am sites were identified as read starts that were at least 2-fold enriched in m6ACE libraries than in the corresponding input libraries. This enrichment was calculated using DESeq2^52^ performed on A-only sites across triplicate pairs of m6ACE and corresponding input libraries (FDR < 0.1, padj < 0.05). Based on read-start patterns observed from m6ACE-seq of methylated spike-ins, we considered identified sites that were 1–4 nucleotides upstream of another identified significant Rm6AC site or sites found within clustered read-starts to be m6ACE-seq false-positives and filtered them out. To identify m6A or m6Am sites that were differentially methylated between sample conditions, we calculated the RML of each site in each sample: The read-start counts at positions −4 to 0 of each site in the m6ACE library were summed and divided by the read-start counts at positions −51 to 0 of the same site in the input library to give ‘X’. Similarly, the read-start counts at positions −4 to 0 of the spike-in m6A site in the m6ACE library were summed and divided by the read-start counts at positions −21 to 0 of the same spike-in m6A site in the input library to give ‘Y’. X was normalised to Y to give RML. RML values of each site was averaged across triplicates for each sample condition. A site was denoted as differentially methylated if the average RML differs between sample conditions with a log_2_fold-change (LFC) cutoff of 2.0 (for methylase-KO or demethylase-OE induced RML reduction) or 1.0 (for demethylase-KO induced RML accumulation), as well as a one-tailed Student’s *T*-test *p*-value cutoff of < 0.05. Consensus motif analysis was performed using Meme-chip^53^. Metagene analysis was performed using MetaPlotR^54^. Gene ontology analysis was performed using the PANTHER classification system^55^. Probability of overlap of lists of m6A/m6Am sites were calculated using a hypergeometric distribution. ROCAUC analysis was performed as described^56^ with the following changes: the collection of all m6A and m6Am sites present in WT cells or exhibiting RML accumulation in demethylase-KO cells were ranked with the most insignificant site first, based on WT padj-value as calculated by DESeq2. An ROC curve was plotted based on the ability for a demethylase-regulated site (at LFC = 0.0, 0.5, 1.0, 1.5; *T*-test *p* < 0.05) to predict insignificant m6A/m6Am sites in WT cells, and the area under the curve was calculated.

### T3 DNA ligase assay for m6A detection

100 ng of DNAse-treated RNA was annealed with respective pairs of DNA probes (Supplementary Data 1) in T3 DNA ligase buffer [66 mM Tris pH 7.6, 10 mM MgCl_2_, 1 mM ATP, 1 mM DTT, 7.5% PEG6000] by incubating for 3 min at 85 °C and 10 min at 35 °C. 1U of T3 DNA ligase was added and the reaction incubated for 15 min at 35 °C. DNA probe ligation efficiency was quantified using quantitative PCR (qPCR) as described below.

### RT-qPCR

Reverse transcription with real-time qPCR (RT-qPCR) was performed as described: Briefly, total RNA was treated with RQ1 DNAse (Promega M610A) according to manufacturer’s instructions. RNA was then purified using Phenol-chloroform-Isoamyalcohol (25:24:1) and precipitated with ethanol. Purified RNA was incubated with 125 ng of random hexamers and 0.5 μl of 10 mM dNTPs for 65 °C at 5 mins and placed on ice for at least 1 min. Reverse transcription was performed with 200U of superscript III (Invitrogen 18080044) and incubated for 25 °C at 5 min, 50 °C at 1 h and 70 °C at 15 min in a 20-μl reaction. 5U of RNase H (Invitrogen 18020171) was added to each sample to digest remaining RNA for 20 mins at 37 °C. cDNA was used for RT-qPCR with express SYBR greenER qPCR supermix (Invitrogen 11762100), according to manufacturer’s instructions, and with respective forward and reverse primers as listed (Supplementary Data 1).

### Total protein isolation

Trypsinized HEK293T cells were washed twice with ice-cold PBS. Washed cells or intact nuclei were lysed in RIPA buffer [150 mM NaCl, 1% NP-40, 0.5% sodium deoxycholate 0.1% SDS, 50 mM Tris pH 8, 1X Complete Mini EDTA-free protease inhibitor] by tumbling for 30 mins at 4 °C. Lysate was clarified by centrifuging at 16,000 × *g* for 30 mins at 4 °C and protein concentration was quantified by Pierce BCA protein assay kit (Thermo Scientific 23225).

### Western blotting

Western blotting was performed as described^31^. Briefly, whole cell or nuclear lysates were diluted in 1x Laemmli buffer (Biorad 1610747) supplemented with 2-mercaptoethanol, before being denatured for 10 min at 95 °C. ~30 µg lysate was separated on a 10% SDS-PAGE gel in separation buffer [25 mM Tris pH 8.3, 192 mM glycine, 0.1% SDS] and transferred onto a nitrocellulose membrane via wet transfer in cold transfer buffer [25 mM Tris pH 8.3, 192 mM glycine, 20% methanol]. The membrane was rinsed with water then blocked with Odyssey blocking buffer (Licor 927) for 1 h at room temperature. Membrane was stained for 16 hr at 4 °C overnight with antibody dilution solution [0.1% Tween-20 in Odyssey blocking buffer] containing primary antibodies. Membrane was rinsed then washed thrice with PBS-T [1X PBS, 0.1% Tween20], before being stained with secondary antibodies. Membrane was rinsed then washed thrice with PBS-T and once with PBS before being imaged on a Licor Odyssey CLx imaging system. The following antibodies and dilution scales were used: 1 µg ml^−1^ Mouse anti-actin (Santa Cruz sc-8432); 200x diluted mouse anti-HSP60 (Abcam ab110312); 1000x diluted rabbit anti-METTL3 (Bethyl Lab A301-567A-T), 1000x diluted rabbit anti-METTL16 (Bethyl Lab A304-192A-T), 1000x diluted rabbit anti-PCIF1 (Bethyl Lab A304-711A-T), 250x diluted rabbit anti-ALKBH5 (Sigma HPA007196), 1000x diluted rabbit anti-FTO (Abcam ab126605), 1000x diluted rabbit anti-CALNEXIN (Abcam ab22595), 2000x diluted mouse anti-TBP (Abcam ab818), 2000x diluted rabbit anti-NCBP2 (Abcam ab91560), 10,000x diluted IRDye 680RD goat anti-mouse IgG H + L (Licor 68070) and 10,000x diluted IRDye 800CW goat anti-rabbit IgG H + L (Licor 32211).

### Immunofluorescence

Cells were seeded on poly-d-lysine coated coverslips (Neuvitro GG-14-PDL) in 24-well plates 24–48 h before fixing. At ~70% confluency, cells were washed in PBS once and fixed in 4% formaldehyde (diluted in PBS, Thermo 28906) for 10 mins. The cells were rinsed and washed thrice using cold PBS. All washes steps were done in 0.5 mL volume with shaking at 50 rpm for 5 mins at room temperature unless otherwise stated. Permeabilization of cell membranes was done using 1% Triton X-100 (diluted in PBS) for 10 mins. The cells were rinsed and washed thrice using PBS-T [PBS with 0.1% Tween 20]. Blocking was then done using PBS-T with 10% goat serum (Sigma G9023) for 1 hr at RTP. Primary antibodies used are rabbit anti-FTO (anti-FTO i, Abcam ab126605, 1:1000), mouse anti-FTO (anti-FTO ii, Santa Cruz sc271713, 1:100) and mouse anti-FLAG (Sigma F1804, 1:1000), which were diluted in antibody dilution buffer [PBS-T with 1% goat serum]. The blocking solution was aspirated and the diluted primary antibody added directly to coverslip before incubating overnight in a 4 °C humid chamber. After which, the cells were rinsed and washed thrice using PBS-T. Fluorescent secondary antibodies (Invitrogen A11019 & A11070) were also diluted in antibody dilution buffer and added directly to the coverslip before incubation in the dark for 1 hr at room temperature. The cells were rinsed and washed thrice using PBS-T in the dark. Hoechst solution was prepared by diluting Hoechst 33342 (Invitrogen H3570) in PBS. 2 µg ml^−1^ of Hoechst solution was used for nuclear staining in the dark for 5 mins. The cells were rinsed and washed thrice in PBS in the dark for 10 mins each. The coverslips were then placed onto a drop of Prolong Diamond Antifade Mountant (Thermo P36970) on a glass slide, cured overnight and sealed with nail polish. Images were taken with a Leica DMi8 microscope.

### snRNA isolation and nucleoside UHPLC-MS/MS

Nuclear-enriched RNA was resolved on a 6% TBE-urea gel then stained with SYBR-gold (Invitrogen S11494). U1/5.8s-rRNA and U4 snRNAs were purified by cutting out the 164nt and 141nt bands respectively, using the low-range ssRNA ladder as a size marker (NEB N0364). snRNAs were gel eluted in elution buffer [0.4 M NaCl, 10 mM Tris pH 7.5, 1 mM EDTA pH 8] at 16 °C overnight with shaking at 2000 rpm. RNA was then precipitated with equal volume isopropanol and washed with 70% ethanol before the pellet was dissolved in water. Up to 100 ng of each snRNA was decapped in a 10 µl reaction with 1x ThermoPol buffer (NEB B9004S) and 5U RppH (NEB M0356) for 2 hr at 37 °C. The reaction was supplemented with 2 µl 1U µl^−1^ Nuclease P1 (Sigma N8630) in a 30-µl reaction with 0.2 mM ZnCl_2_ and 20 mM NH_4_OAc pH 5.3, incubated for 2 hr at 42 °C. The reaction was finally supplemented with 2 µl 1 mU µl^−1^ phosphodiesterase (Sigma P3242) and 2 µl 1U µl^−1^ alkaline phosphatase (Sigma P5931) in a 40-µl reaction with 100 mM NH_4_HCO_3_, incubated for 2 h at 37 °C, then heat inactivated for 5 min at 65 °C and subjected to UHPLC-MS/MS.

Nucleoside UHPLC-MS/MS was performed at the Singapore Phenome Centre as previously described^31^. Briefly, for reverse phase liquid chromatograph, a HSS T3 (1.8 µm; 2.1 × 100 mm) column was used with the following parameters. Mobile phase A: water + 0.1% formic acid; mobile phase B: acetonitrile + 0.1% formic acid; flow rate: 0.3 ml min^−1^; column temperature: 40 °C; sample temperature: 4 °C; injection volume: 5 µl; sample loop: 5 µl. Elution gradient condition was set as 0 min 2%B, 0.5 min 2%B, 6 min 8%B, 6.5 min 8%B, 6.6 min 2%B, 8 min 2%B. Am and m6Am eluted with the retention times of 3.64 min and 5.64 min respectively. Tandem mass spectrometry was performed using a Xevo TQ-S, Waters machine with the following parameters. Ion mode: ESI positive; Acquisition model: MRM; capillary voltage: 3.2 kV; desolvation temperature: 400 °C; Con gas flow: 150 L h^−1^; desolvation gas flow: 800 L h^−1^; source temperature: 150 °C; collision energy: 16 eV. Am and m6Am were detected by monitoring mass transitions of m z^−1^ = 282.27 → 136.0 and m z^−1^ = 296.0 → 150.0 respectively. Sample Am or m6Am were quantified based on a linear calibration curve generated using Am (Berry & Associates PR3734) or m6Am (Carbosynth NM157470) nucleoside standards. All measurements were performed in technical triplicates.

### Reporting summary

Further information on research design is available in the Nature Research Reporting Summary linked to this article.

## Supplementary information

Supplementary Information

Reporting Summary

Description of Additional Supplementary Files

Supplementary Data 1

Supplementary Data 2

Supplementary Data 3

Supplementary Data 4

Supplementary Data 5

Supplementary Data 6

Supplementary Data 7

Supplementary Data 8

Supplementary Data 9

Supplementary Data 10

## Data Availability

A reporting summary for this Article is available as a Supplementary Information file. Sequencing data were deposited in NCBI’s Gene Expression Omnibus (GEO) under accession number GSE124509. All data is available from the corresponding author upon reasonable request.

## Code availability

The custom Python scripts used in this study are available on request from the corresponding author.
