# Supplementary material for: Atlas of quantitative single-base-resolution N6-methyl-adenine methylomes
Source: Nat Commun. 2019 Dec 10;10:5636. doi: 10.1038/s41467-019-13561-z (PMC6904561; doi:10.1038/s41467-019-13561-z)
Supplement: Supplementary file 1 — Supplementary Information [file 41467_2019_13561_MOESM1_ESM.pdf]

Koh *et al.* Supplementary Information

An atlas of single-base-resolution N^6-methyl-adenine methylomes suggests RNA demethylases as suppressors of RNA methylation

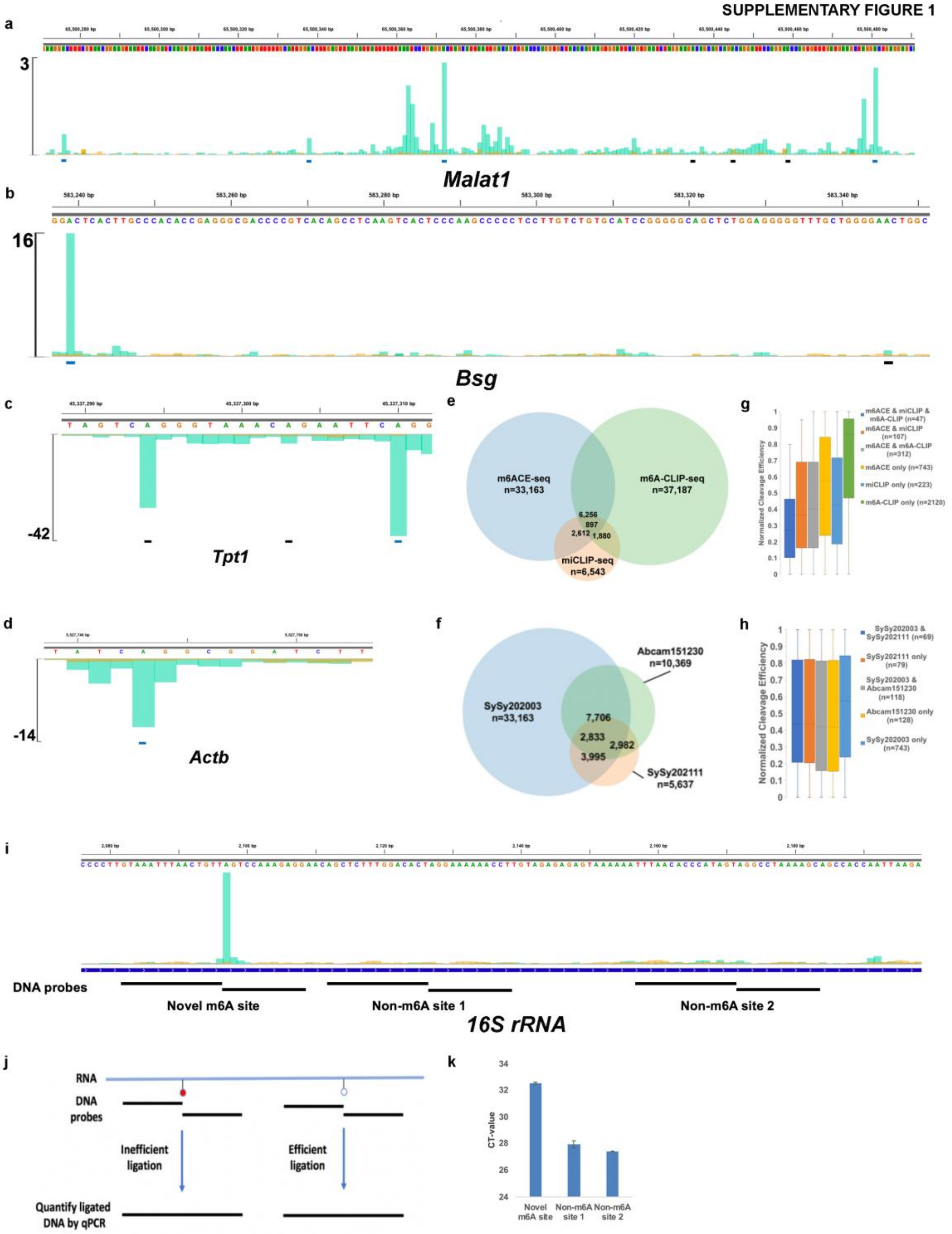

Supplementary Figure 1. Validation of m6ACE-seq.

(a-d) m6ACE (green) overlaid on Input (orange) read-start RPM counts mapped to genes previously validated by SCARLET[1]. Sequence corresponds to the same strand as the m6A site. Blue and black horizontal bars denote SCARLET positive and negative sites respectively.

(e) Overlap in sites identified using m6ACE-seq of HEK293T RNA, miCLIP-seq of HEK293 RNA[2] and m6A-CLIP-seq of HeLa cytoplasmic RNA[3]. Hypergeometric test for significance of overlap $p<10^{-100}$.

(f) Overlap in sites identified using m6ACE-seq performed with the main m6A-specific antibody versus m6ACE-seq performed with SynapticSystems202111 or Abcam151230. Hypergeometric test for significance of overlap $p<10^{-100}$.

(g,h) Normalized cleavage efficiency, which was defined as an inverse stoichiometric quantitation of m6A methylation level and only applies to sites within a "m6ACA" motif, was downloaded from a public database[4]. Displayed are normalized cleavage efficiencies of m6A sites identified using either different m6A-sequencing methods (g) or using m6ACE-seq with different antibodies (h). Centre line, box boundaries, lower whisker and upper whisker represent the median, interquartile range, minimum value and maximum value respectively.

(i) m6ACE (green) overlaid on Input (orange) read-start RPM counts mapped to mitochondrial 16S rRNA. Sequence corresponds to the same strand as the m6A site. Blue horizontal bar represents the 16S rRNA transcript. DNA probes used in T3 DNA ligase assay are also represented.

(j) Schematic outlining T3 DNA ligase assay for m6A detection[5]. Filled and empty circles represent 'm6A' and 'A' respectively.

(k) qPCR CT-values as an inverse measure of ligation efficiency of DNA probes depicted in (i). Represented are averages of technical triplicates with error bars (s.d.).

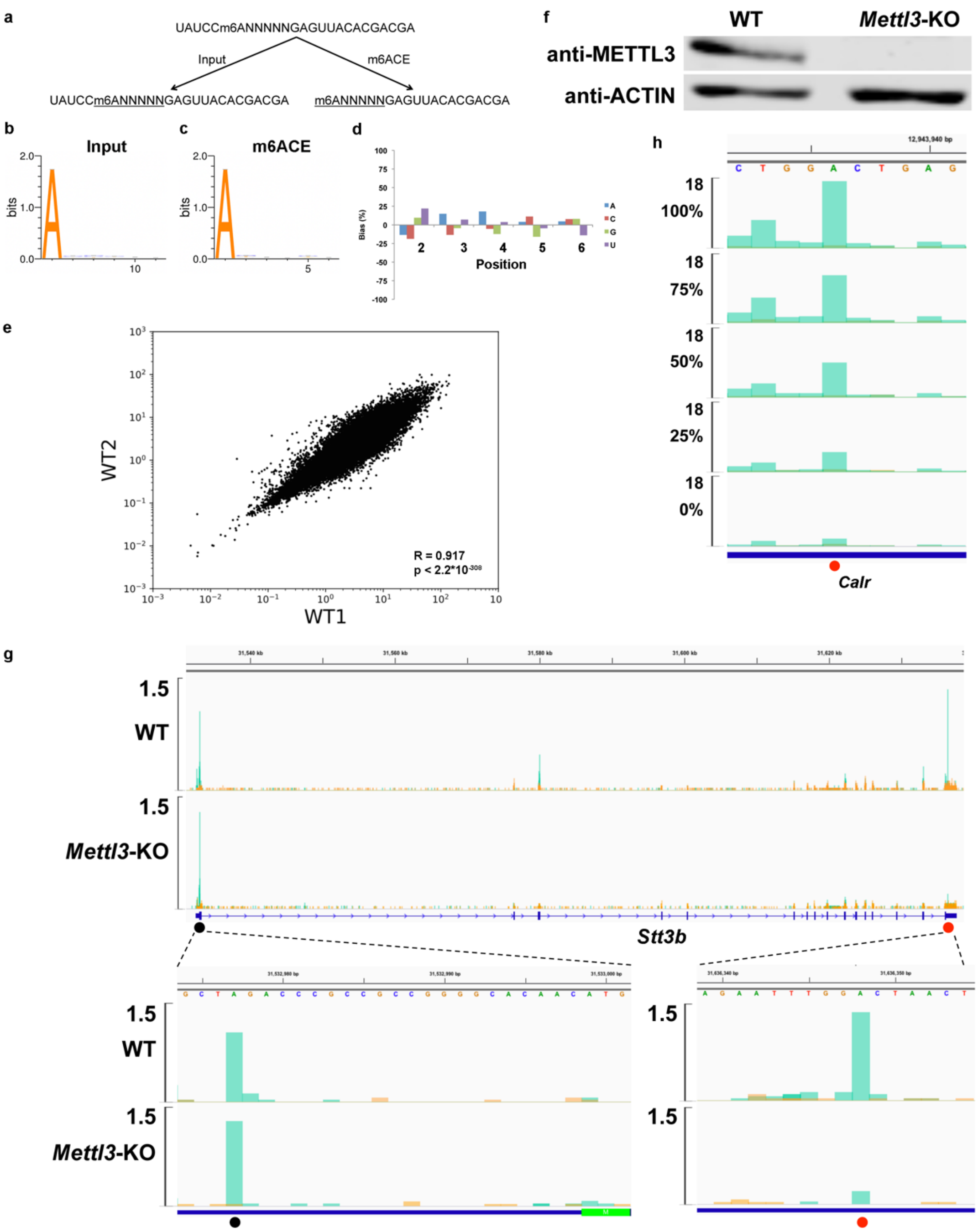

Supplementary Figure 2. m6ACE-seq maps RML reductions at individual METTL3-dependent m6A.

(a) Strategy to determine if m6A in the first RNA position causes any sequence bias in the subsequent sequences. A synthetic RNA oligonucleotide containing an internal m6A followed by 5 random sequences (Supplementary Data 1) is subjected to m6ACE-seq. m6ACE treatment will remove the first 5 nucleotides. Ligation bias can by calculated as the enrichment of underlined sequences in the m6ACE library (nt1-6) over that of the input library

(nt6-11). (b,c) Sequence bias at nt6-11 in the input library (b) and nt1-6 in the m6ACE library (c) as calculated using Weblogo[6].

(d) Ligation bias of each nucleotide in nt2-6 of the m6ACE library over nt7-11 in the input library. Positive and negative percentages represent nucleotides that are respectively biased for or against in the m6ACE library. For example, "-100%" indicates a nucleotide that is completely excluded in the m6ACE library compared to the input library.

(e) Scatterplot of average RML of a first set of WT triplicate samples versus a second set of WT triplicate samples. R-value and p-value of linear regression fit of the 2 sets of average RMLs are reported.

(f) Western blotting demonstrating complete loss of METTL3 in *Mettl3*-KO cells.

(g,h) m6ACE (green) overlaid on Input (orange) read-start RPM counts mapped to a representative gene. METTL3-dependent and independent sites are respectively denoted by a red or black dot. Sequence corresponds to the same strand as the m6A site. Blue horizontal bars represent transcript models, with the green sections representing the start codon. Magnified views of the 5'UTR and 3'UTR are also displayed for (g). For (h), percentages represent proportion of WT RNA in a mixture of WT and *Mettl3*-KO RNA.

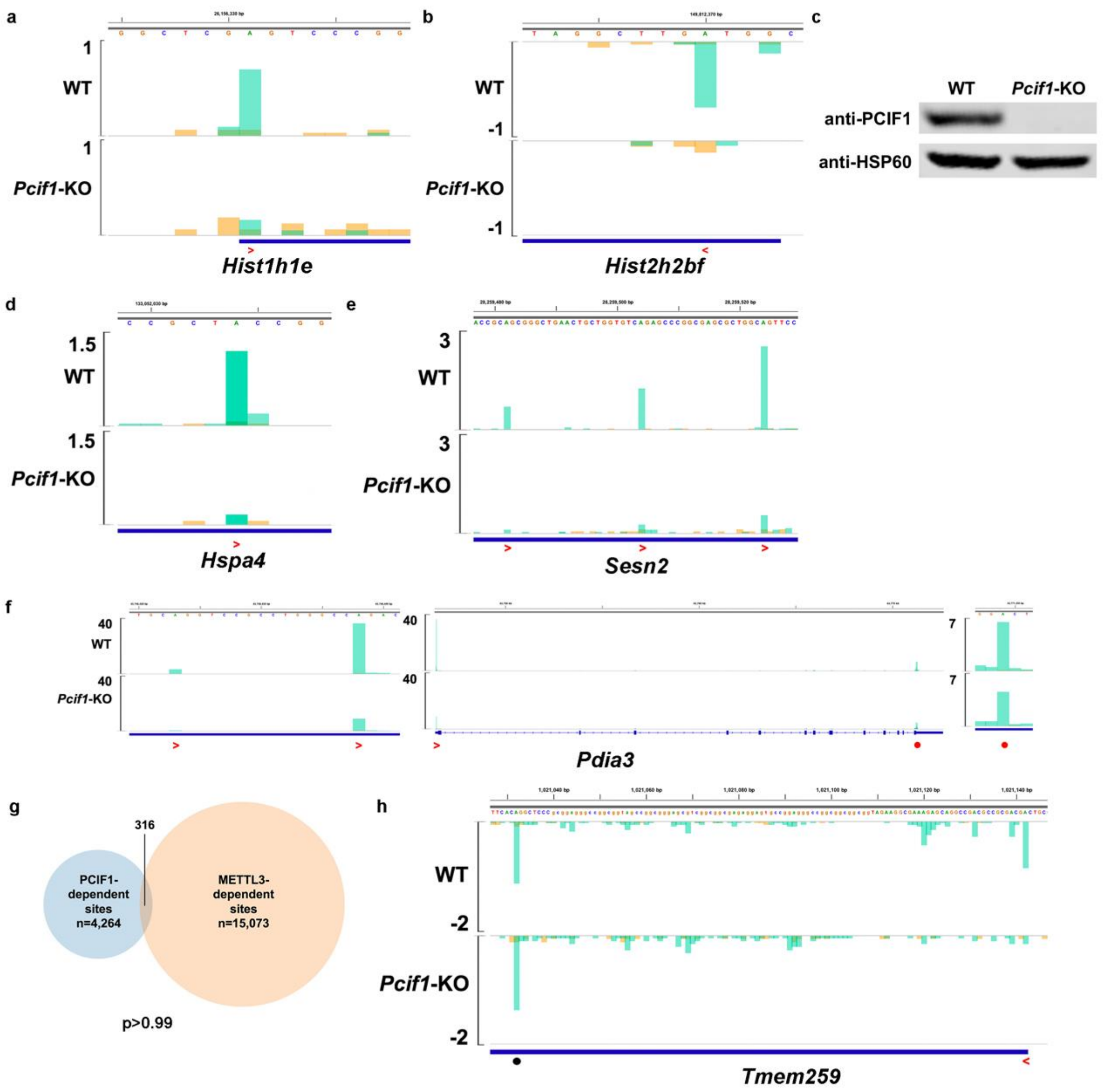

Supplementary Figure 3. m6ACE-seq maps RML reductions at individual PCIF1-dependent m6Am sites.

(a,b,d,e,f,h) m6ACE (green) overlaid on Input (orange) read-start RPM counts mapped to a representative 5'UTRs. PCIF1-dependent, PCIF1-independent and METTL3-dependent sites are respectively denoted by red angles, black dots and red dots. Sequence corresponds to the same strand as the m6A/m6Am sites. Blue horizontal bars represent transcript models. Magnified views of the 5'UTR and 3'UTR are also displayed for (f). PCIF1-independent sites are sites where average RML reduction from WT to PCIF-KO is not significantly more than 1.5 fold (Student's t-test p<0.05).

(c) Western blotting demonstrating loss of PCIF1 in *Pcif1*-KO cells.

(g) Venn diagram representing overlap between PCIF1-dependent and METTL3-dependent sites. p-value of overlap by chance was calculated with a hypergeometric test.

SUPPLEMENTARY FIGURE 4

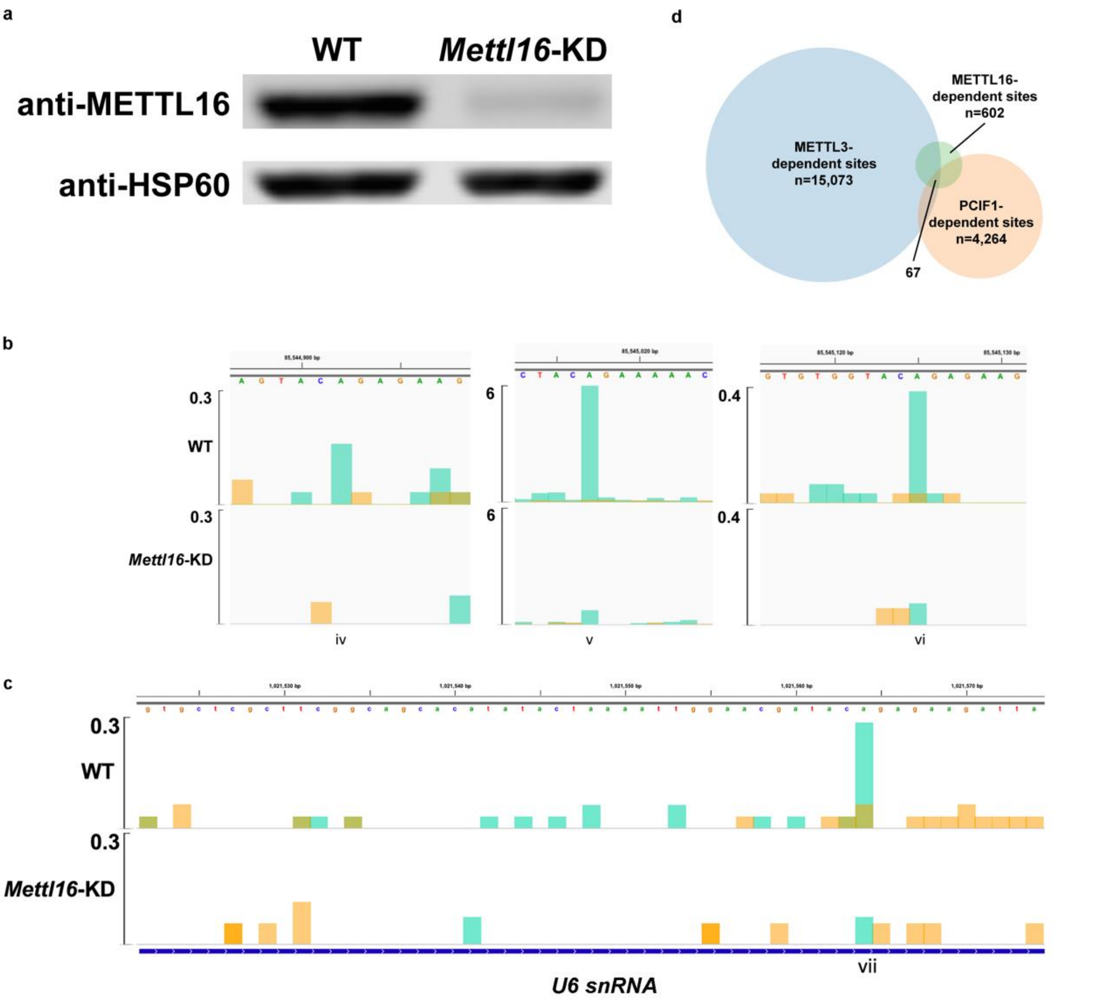

Supplementary Figure 4. m6ACE-seq maps METTL16-dependent RML reductions at 'UACAGAGAA' sites within the *Mat2a* 3'UTR.

(a) Western blotting demonstrating depletion of METTL16 in *Mettl16*-KD cells.

(b) Magnified representations of the *Mat2a* 3'UTR at positions iv, v and vi as denoted in (Fig. 4a).

(c) m6ACE (green) overlaid on Input (orange) read-start RPM counts mapped to *U6* snRNA. Sequence corresponds to the same strand as the m6A site, denoted by position vii. Blue horizontal bars represent *U6 snRNA* transcript.

(d) Venn diagram representing overlap between METTL16-dependent with METTL3-dependent and PCIF1-dependent sites.

SUPPLEMENTARY FIGURE 5

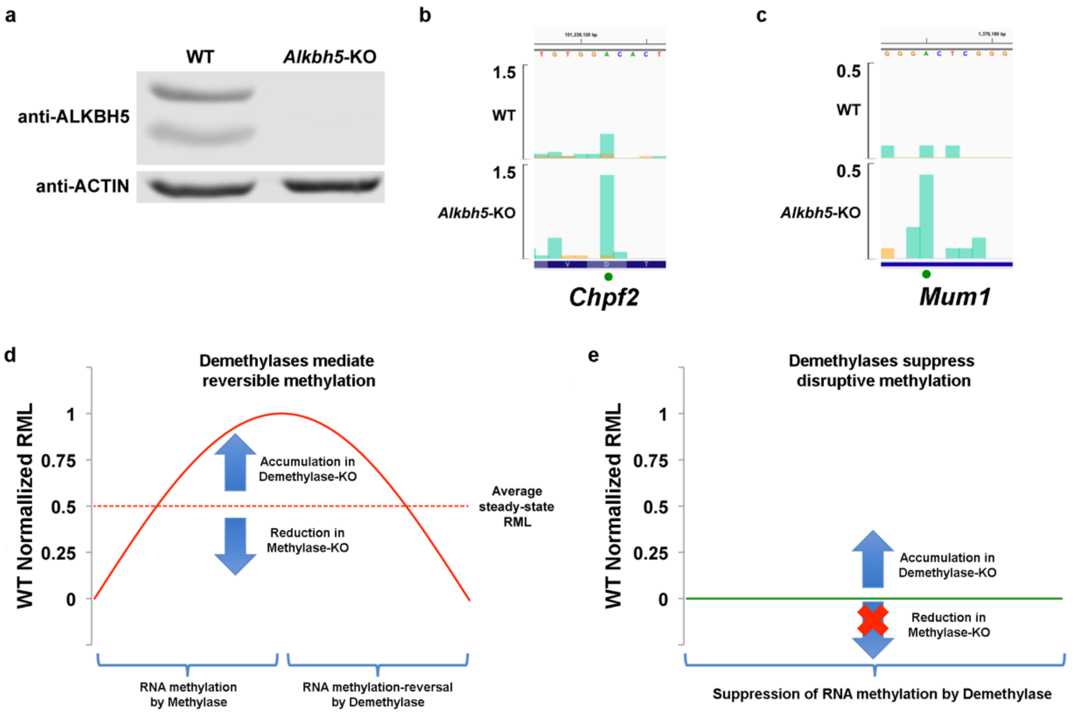

Supplementary Figure 5. ALKBH5 demethylates m6A in vivo.

(a) Western blotting demonstrating complete loss of ALKBH5 in *Alkbh5*-KO cells.

(b,c) m6ACE (green) overlaid on Input (orange) read-start RPM counts mapped to a representative CDS (b) or 3’UTR (c). ALKBH5-regulated sites are denoted by green dots. Sequence corresponds to the same strand as the m6A sites. Blue horizontal bars represent transcript models.

(d,e) Simplified models for expected RML variations given a model where ALKBH5 mediates reversible RNA methylation in the cytoplasm (d) versus a model where ALKBH5 suppresses methylation from accumulating in the nucleus (e).

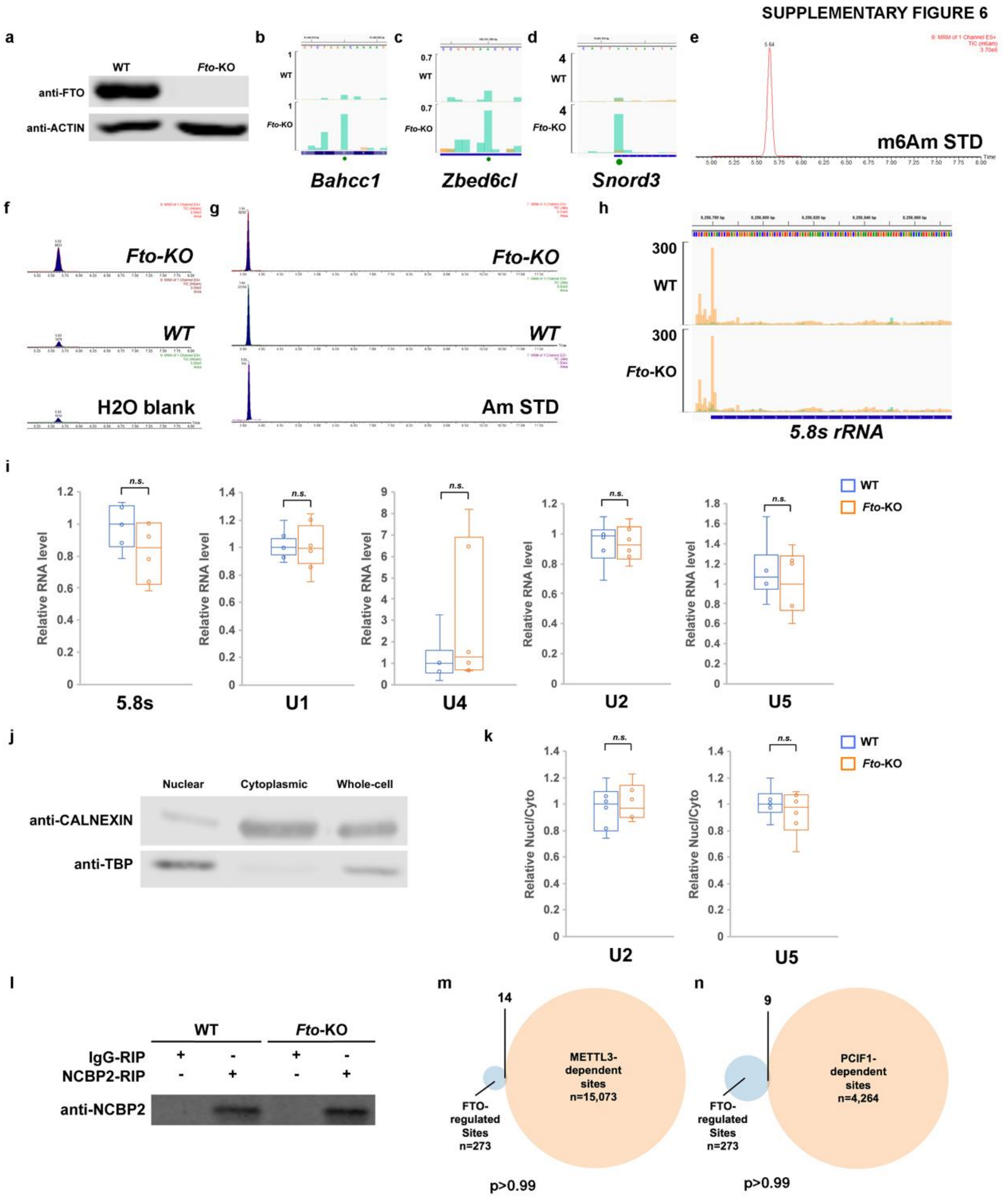

Supplementary Figure 6. FTO suppresses accumulation of disruptive methylation in specific RNAs.

(a) Western blotting demonstrating complete loss of FTO in *Fto*-KO cells.

(b-d,h) m6ACE (green) overlaid on Input (orange) read-start RPM counts mapped to a representative CDS (b), 3’UTR (c) or sRNAs (d,h). FTO-regulated sites are denoted by green dots. Sequence corresponds to the same strand as the m6A/m6Am sites. Blue horizontal bars represent transcript models.

(e) Chromatogram of m6Am standard with retention time denoted.

(f,g) Chromatogram of m6Am (f) or Am (g) with peak areas and retention times denoted. WT m6Am amounts are similar to that in H2O blanks and are thus in the non-quantifiable range (f).

(i) Box and whisker plots of various sRNA total levels relative to *7SL scRNA* in WT (blue) and *Fto*-KO (orange) cells. Represented are median and interquartile ranges for 6 biological replicates, normalized to the corresponding median values in WT cells.

(j) Western blotting demonstrating enrichment of TBP (nuclear marker) and de-enrichment of CALNEXIN (endoplasmic reticulum/cytoplasmic marker) in nuclear lysate, as well as de-enrichment of TBP and enrichment of CALNEXIN in cytoplasmic lysate compared to whole-cell lysate.

(k) Box and whisker plots of ratio of sRNA levels (relative to *7SL scRNA*) in nucleus over cytoplasm in WT (blue) and *Fto*-KO (orange) cells. Represented are median and interquartile ranges for 6 biological replicates, normalized to the corresponding median values in WT cells.

(l) Western blotting demonstrating specific pulldown of NCBP2 protein by anti-NCBP2 but not non-specific rabbit IgG antibody in both WT and *Fto*-KO cells.

(m,n) Venn diagrams representing overlaps between FTO-regulated sites with METTL3-dependent m6A (m) or PCIF1-dependent m6Am (n). p-value of overlap by chance was calculated with a hypergeometric test.

n.s. denotes not significant. For box and whisker plots, centre line, box boundaries, lower whisker and upper whisker represent the median, interquartile range, minimum value and maximum value respectively.

References

1. Liu, N. *et al.* Probing N6-methyladenosine RNA modification status at single nucleotide resolution in mRNA and long noncoding RNA. *RNA* **19,** 1848–1856 (2013).
2. Linder, B. *et al.* Single-nucleotide-resolution mapping of m6A and m6Am throughout the transcriptome. *Nature Methods* **12,** 767–772 (2015).
3. Ke, S. *et al.* m6A mRNA modifications are deposited in nascent pre-mRNA and are not required for splicing but do specify cytoplasmic turnover. *Genes & Development* **31,** 990–1006 (2017).
4. Garcia-Campos, M. A. *et al.* Deciphering the ‘m6A Code’ via Antibody-Independent Quantitative Profiling. *Cell* (2019). doi:10.1016/j.cell.2019.06.013
5. Liu, W. *et al.* Identification of a selective DNA ligase for accurate recognition and ultrasensitive quantification of N6-methyladenosine in RNA at one-nucleotide resolution. *Chemical Science* **9,** 3354–3359 (2018).
6. Crooks, G. E., Hon, G., Chandonia, J.-M. & Brenner, S. E. WebLogo: a sequence logo generator. *Genome Research* **14,** 1188–1190 (2004).
