# Supplementary material for: Atlas of quantitative single-base-resolution N6-methyl-adenine methylomes
Source: Nat Commun. 2019 Dec 10;10:5636. doi: 10.1038/s41467-019-13561-z (PMC6904561; doi:10.1038/s41467-019-13561-z)
Supplement: Supplementary file 2 — Reporting Summary [file 41467_2019_13561_MOESM2_ESM.pdf]

nature research

Corresponding author(s): W.S. Sho Goh

Reporting Summary

Nature Research wishes to improve the reproducibility of the work that we publish. This form provides structure for consistency and transparency in reporting. For further information on Nature Research policies, see Authors & Referees and the Editorial Policy Checklist.

Statistical parameters

When statistical analyses are reported, confirm that the following items are present in the relevant location (e.g. figure legend, table legend, main text, or Methods section).

n/a	Confirmed	
☐	☒	The exact sample size (*n*) for each experimental group/condition, given as a discrete number and unit of measurement
☐	☒	An indication of whether measurements were taken from distinct samples or whether the same sample was measured repeatedly
☐	☒	The statistical test(s) used AND whether they are one- or two-sided<br>*Only common tests should be described solely by name; describe more complex techniques in the Methods section.*
☒	☐	A description of all covariates tested
☐	☒	A description of any assumptions or corrections, such as tests of normality and adjustment for multiple comparisons
☐	☒	A full description of the statistics including central tendency (e.g. means) or other basic estimates (e.g. regression coefficient) AND variation (e.g. standard deviation) or associated estimates of uncertainty (e.g. confidence intervals)
☐	☒	For null hypothesis testing, the test statistic (e.g. *F*, *t*, *r*) with confidence intervals, effect sizes, degrees of freedom and *P* value noted<br>*Give P values as exact values whenever suitable.*
☒	☐	For Bayesian analysis, information on the choice of priors and Markov chain Monte Carlo settings
☒	☐	For hierarchical and complex designs, identification of the appropriate level for tests and full reporting of outcomes
☒	☐	Estimates of effect sizes (e.g. Cohen's *d*, Pearson's *r*), indicating how they were calculated
☐	☒	Clearly defined error bars<br>*State explicitly what error bars represent (e.g. SD, SE, CI)*

Our web collection on statistics for biologists may be useful.

Software and code

Policy information about availability of computer code

Data collection	Illumina sequencing base calls were performed using FASTQ Generation v1.0.0. For nucleoside UHPLC-MS/MS, MassLynx was used for chromatography and TargetLynx for quantification. For Western blotting, images were captured using ImageStudioLite.
Data analysis	Fastq sequences were first trimmed of 5' and 3' adapter sequences and poly(A) tails using Cutadapt. The 8-mer 'N7B' (N=A/C/G/T, B=C/G/T) UMI located at the first 8 nucleotides of read 1 was registered and trimmed. Any complementary UMI sequence in read 2 was also trimmed. Reads were mapped to the methylated spike-in (Supplementary Data 1) using Bowtie2, or to the hg38 assembly transcriptome (Gencode v28 comprehensive gene annotations) using STAR. Aligned pairs that had the same mapping coordinates and UMIs were filtered out as PCR duplicates. Read-start coordinates in hg38-mapped reads that began with an adenosine nucleotide, and had a minimum mean read count of 1 across the triplicate samples were collated. m6A or m6Am sites were identified as read starts that were at least 2-fold enriched in m6ACE libraries than in the corresponding input libraries. This enrichment was calculated using DESeq2 performed on A-only sites across triplicate pairs of m6ACE and corresponding input libraries (FDR<0.1, padj<0.05). Based on read-start patterns observed from m6ACE-seq of methylated spike-ins, we considered identified sites that were 1-4 nucleotides upstream of another identified significant Rm6AC site or sites found within clustered read-starts to be m6ACE-seq false-positives and filtered them out. To identify m6A or m6Am sites that were differentially methylated between sample conditions, we calculated the RML of each site in each sample: The read-start counts at positions -4 to 0 of each site in the m6ACE library were summed and divided by the read-start counts at positions -51 to 0 of the same site in the input library to give 'X'. Similarly, the read-start counts at positions -4 to 0 of the spike-in m6A site in the m6ACE library were summed and divided by the read-start counts at positions -21 to 0 of the same spike-in m6A site in the input library to give 'Y'. X was normalized to Y to give RML. RML values of each site was averaged across triplicates for each sample condition. A site was denoted as differentially methylated if the average RML differs between sample conditions with a log2fold-change

(LFC) cutoff of 2.0 (for methylase-KO or demethylase-OE induced RML reduction) or 1.0 (for demethylase-KO induced RML accumulation), as well as a one-tailed T-test p-value cutoff of <0.05. Consensus motif analysis was performed using Meme-chip. Metagene analysis was performed using MetaPlotR. Gene ontology analysis was performed using the PANTHER classification system. Probability of overlap of lists of m6A/m6Am sites were calculated using a hypergeometric distribution. ROCAUC analysis was performed as previously described with the following changes: the collection of all m6A and m6Am sites present in WT cells or exhibiting RML accumulation in demethylase-KO cells were ranked with the most insignificant site first, based on WT padj-value as calculated by DESeq2. An ROC curve was plotted based on the ability for a demethylase-regulated site (at LFC=0.0,0.5,1.0,1.5; T-test p<0.05) to predict insignificant m6A/m6Am sites in WT cells, and the area under the curve was calculated.

For manuscripts utilizing custom algorithms or software that are central to the research but not yet described in published literature, software must be made available to editors/reviewers upon request. We strongly encourage code deposition in a community repository (e.g. GitHub). See the Nature Research guidelines for submitting code & software for further information.

Data

Policy information about availability of data

All manuscripts must include a data availability statement. This statement should provide the following information, where applicable:
- Accession codes, unique identifiers, or web links for publicly available datasets
- A list of figures that have associated raw data
- A description of any restrictions on data availability

Data were deposited in NCBI's Gene Expression Omnibus (GEO) under accession number GSE119094.

Field-specific reporting

Please select the best fit for your research. If you are not sure, read the appropriate sections before making your selection.

[x] Life sciences [ ] Behavioural & social sciences [ ] Ecological, evolutionary & environmental sciences

For a reference copy of the document with all sections, see nature.com/authors/policies/ReportingSummary-flat.pdf

Life sciences study design

All studies must disclose on these points even when the disclosure is negative.

Sample size	We used a minimum of biological triplicates.
Data exclusions	No data was excluded.
Replication	Experiments were repeated to verify reproducibility.
Randomization	This is not relevant as we worked with cell lines.
Blinding	Blinding was not relevant in our studies.

Reporting for specific materials, systems and methods

Materials & experimental systems

n/a	Involved in the study
[ ]	[x] Unique biological materials
[ ]	[x] Antibodies
[ ]	[x] Eukaryotic cell lines
[x]	[ ] Palaeontology
[x]	[ ] Animals and other organisms
[x]	[ ] Human research participants

Methods

n/a	Involved in the study
[x]	[ ] ChIP-seq
[x]	[ ] Flow cytometry
[x]	[ ] MRI-based neuroimaging

Unique biological materials

Policy information about availability of materials

Obtaining unique materials	Gene-knockout and knockdown cells were generated using CRISPR-Cas9 procedures or siRNA knockdown respectively.

Antibodies

Antibodies used	anti-m6A (Synaptic Systems 202003)<br>anti-m6A (Synaptic Systems 202111)<br>anti-m6A (Abcam 151230)<br>anti-actin (Santa Cruz sc-8432)<br>anti-HSP60 (Abcam ab110312)<br>anti-METTL3 (Bethyl Lab A301-567A-T)<br>anti-METTL16 (Bethyl Lab A304-192A-T)<br>anti-PCIF1 (Bethyl Lab A304-711A-T)<br>anti-ALKBH5 (Sigma HPA007196)<br>anti-FTO (Abcam ab126605)<br>anti-FTO (Santa Cruz sc271713)<br>anti-CALNEXIN (Abcam ab22595)<br>anti-TBP (Abcam ab818)<br>anti-NCBP2 (Abcam ab91560)<br>IRDye 680RD goat anti-mouse IgG H+L (Licor 68070)<br>IRDye 800CW goat anti-rabbit IgG H+L (Licor 32211)<br>F(ab')2-Goat anti-Mouse IgG (H+L) Cross-Adsorbed Secondary Antibody, Alexa Fluor 568 (Invitrogen A-11019)<br>F(ab')2-Goat anti-Rabbit IgG (H+L) Cross-Adsorbed Secondary Antibody, Alexa Fluor 488 (Invitrogen A-11070)
Validation	Antibodies were validated either by observing for a loss of the correct size Western blotting protein band using the respective gene-KO cell lines or by the manufacturers.

Eukaryotic cell lines

Policy information about cell lines

Cell line source(s)	ATCC HEK293T CRL-3216
Authentication	ATCC HEK293T CRL-3216 was authenticated via ATCC STR profiling.
Mycoplasma contamination	HEK293T cells designated for any genomic extractions were always subjected to MycoAlert Plus Mycoplasma kit (Lonza LT07) and occasionally to EZ-PCR Mycoplasma Detection kit (Biological Industries 20-700-20) to verify that they were mycoplasma-free.
Commonly misidentified lines<br>(See ICLAC register)	*Name any commonly misidentified cell lines used in the study and provide a rationale for their use.*
