# Supplementary material for: Atlas of quantitative single-base-resolution N6-methyl-adenine methylomes
Source: Nat Commun. 2019 Dec 10;10:5636. doi: 10.1038/s41467-019-13561-z (PMC6904561; doi:10.1038/s41467-019-13561-z)
Supplement: Supplementary file 3 — Description of Additional Supplementary Files [file 41467_2019_13561_MOESM3_ESM.docx]

**Description of Additional Supplementary Files**

File name: Supplementary Data 1.

Description: List of oligonucleotides used in this study.

File name: Supplementary Data 2.

Description: RML of sysy202003-m6ACE-seq sites versus sysy202111-m6ACE-seq sites.

File name: Supplementary Data 3.

Description: RML of sysy202003-m6ACE-seq sites versus abcam151230-m6ACE-seq sites.

File name: Supplementary Data 4.

Description: RML of METTL3-dependent sites in wild type and *Mett!3*-KO RNA.

File name: Supplementary Data 5.

Description: RML of METTL3-dependent sites in various mixtures of wild type and *Mett!3*-KO RNA.

File name: Supplementary Data 6.

Description: RML of PCIF1-dependent sites in wild type and *Pcif1*-KO RNA.

File name: Supplementary Data 7.

Description: RML of METTL16-dependent sites in wild type and *Mett!16*-KD RNA.

File name: Supplementary Data 8.

Description: RML of ALKBH5-regulated sites in wild type and *A!kbh5*-KO RNA.

File name: Supplementary Data 9.

Description: RML of FTO-regulated sites in wild type and *Fto*-KO RNA.

File name: Supplementary Data 10.

Description: RML of sites aberrantly affected by FTO in wild type and *Fto*-OE RNA.
